# Intracoronary Structural-Molecular Imaging for Multitargeted Characterization of High-Risk Plaque: First-in-Human OCT-FLIm

**DOI:** 10.1001/jamacardio.2025.0928

**Published:** 2025-05-07

**Authors:** Sunwon Kim, Hyeong Soo Nam, Dong Oh Kang, Jeongmoo Han, Hyokee Kim, Joon Woo Song, Eun Jin Park, Ryeong Hyun Kim, Hyun Jung Kim, Jin Hyuk Kim, Sunki Lee, Young Su Kim, Pyoungjae Park, Man-Jong Baik, Hongki Yoo, Jin Won Kim

**Affiliations:** 1 Multimodal Imaging and Theranostic Lab, Cardiovascular Center, Korea University Guro Hospital, Seoul, South Korea; 2 Department of Cardiology, Korea University Ansan Hospital, Ansan, South Korea; 3 Department of Mechanical Engineering, Korea Advanced Institute of Science and Technology, Daejeon, South Korea; 4 Department of Thoracic and Cardiovascular Surgery, Korea University Guro Hospital, Seoul, South Korea; 5 Division of Transplantation and Vascular Surgery, Korea University Guro Hospital, Seoul, South Korea

## Abstract

**Importance:**

Fluorescence lifetime imaging (FLIm) is a molecular imaging technique used to visualize the biochemical composition of atherosclerosis. Novel dual-modal imaging using optical coherence tomography (OCT)-FLIm has the potential to provide both microstructural and biocompositional information on coronary plaques; however, it needs validation for clinical application.

**Objective:**

To investigate the clinical feasibility and safety of OCT-FLIm for characterizing plaque compositions in patients with coronary artery disease (CAD) undergoing revascularization therapy.

**Design, Setting, and Participants:**

A prospective, open-label, single-center diagnostic feasibility study involving 40 patients with significant CAD requiring coronary revascularization. This first-in-human clinical study of the novel intracoronary OCT-FLIm imaging was conducted between February and August 2022. The analyses were performed from August 2022 to July 2023.

**Interventions:**

An OCT-FLIm system with 2.6-F catheters was constructed. All patients underwent OCT-FLIm for target/culprit and nontarget/nonculprit lesions during coronary revascularization. Intravascular ultrasound imaging was performed for comparison.

**Main Outcomes and Measures:**

The primary outcome was to assess the FLIm-derived molecular readouts of prespecified plaque compositions. The secondary outcome was the feasibility of OCT-FLIm in determining target/culprit plaque compositions across different subsets of atherosclerotic disease activity: (1) acute coronary syndrome (ACS) vs chronic stable angina (CSA) and (2) angiographic rapid disease progression vs nonprogressive controls.

**Results:**

We prospectively enrolled 40 patients (mean [SD] age, 63.1 [8.1] years; 32 men [80.0%]), of whom 20 presented with ACS and 20 with CSA. OCT provided the structural features of plaques, and FLIm characterized the molecular signatures of atheroma compositions, including macrophages, healed plaques, superficial calcification, and fibrosis, in a reproducible manner. Fluorescence lifetime (FL) values of the plaque compositions correlated with findings from prior autopsy studies. Plaque inflammation was significantly greater in patients with ACS than those with CSA. The mean (SD) of inflammation-FL was 7.59 (0.96) nanoseconds for patients with ACS vs 6.46 (0.87) nanoseconds for patients with CSA (*P* < .001). The healed plaque phenotype was more prominently distributed in the segments of rapid disease progression than in nonprogressive controls. The mean (SD) healed plaque-FL was 5.31 (0.20) nanoseconds for the rapidly progressive lesions vs 4.81 (0.30) nanoseconds for the rapidly nonprogressive lesions (*P* < .001). All patients underwent OCT-FLIm safely without adverse clinical events.

**Conclusions and Relevance:**

This diagnostic feasibility study found that an OCT-FLIm structural-molecular intracoronary imaging is clinically feasible and safe for the comprehensive characterization of human atheromas, supporting its potential role in the diagnosis and biological understanding of high-risk plaques.

## Key Points

**Question:**

Can the clinical feasibility and safety of a novel dual-modal intracoronary imaging technique that combines fluorescence lifetime imaging (FLIm) with optical coherence tomography (OCT) be validated for the microstructural and compositional characterization of coronary plaques?

**Findings:**

This first-in-human OCT-FLIm study enrolled 40 patients requiring coronary revascularization. OCT-FLIm characterized the microstructure and molecular signatures of plaque compositions, including macrophages, healed plaques, calcification, and fibrosis, without adverse events and correlated with clinical disease activity across various scenarios.

**Meaning:**

Intravascular OCT-FLIm is clinically feasible and safe for the comprehensive characterization of coronary plaques, offering the potential for advanced assessment of cardiovascular disease.

## Teaser

This diagnostic feasibility study tests whether a novel dual-modal intracoronary imaging technique is a clinically feasible and safe diagnostic tool for identifying high-risk plaques in patients with coronary artery disease.

## Introduction

Coronary artery disease (CAD) accounts for most deaths worldwide. Although intracoronary optical coherence tomography (OCT) can visualize the microstructure of plaques,^1^ given the complex multifactorial pathophysiology of CAD,^2^ it cannot fully explain the biological behavior of plaques. Fluorescence lifetime imaging (FLIm) is an emerging imaging technology that allows the label-free mapping of molecular signatures in biological tissues with high sensitivity and specificity.^3^ Earlier autopsy and preclinical data have shown the ability of FLIm to detect and differentiate the biochemical compositions of atherosclerotic plaques.^4,5,6,7^ Despite the diagnostic potential of FLIm, several technical hurdles still hamper its clinical translation. We developed an OCT-FLIm dual-modal imaging system using a flexible catheter to address these issues.^6^ This first-in-human clinical study aimed to evaluate whether intravascular OCT-FLIm could be used to simultaneously image the microstructure and compositional features of atherosclerotic plaques in coronary arteries and whether FLIm-derived molecular readouts could provide complementary information to characterize plaque phenotypes and their association with disease activity.

## Methods

### OCT-FLIm System and Dual-Modal Catheters

We developed an OCT-FLIm system with a 2.6-F dual-modal imaging catheter for clinical application (eFigure 1 in Supplement 1).^6^ Autofluorescence signals from coronary plaques were separated into 3 distinct spectral bands to measure the corresponding fluorescence lifetimes (FLs): healed plaque-FL, calcification-FL, and inflammation-FL. FL is expressed in nanoseconds for subsequent data visualization and comparative analysis. Detailed technical information is available in the eMethods section in Supplement 1.

### Study Population and Intracoronary Imaging

Between February and August 2022, 40 patients with significant CAD who required coronary revascularization for either acute coronary syndrome (ACS) or chronic stable angina (CSA) were prospectively enrolled (NCT04835467). Each patient underwent OCT-FLIm and intravascular ultrasound (IVUS) of target/culprit and nontarget/nonculprit lesions during revascularization. A *target/culprit lesion* was defined as a plaque requiring revascularization, and a *nontarget/nonculprit lesion* as a plaque with mild to moderate stenosis. OCT-FLIm scans were acquired repeatedly to examine reproducibility. This study was approved by the institutional review board of Korea University Guro Hospital (KUGH; 2021GR0377) and the Korea Ministry of Food and Drug Safety. Written informed consent was obtained from all participants during enrollment. We followed the Standards for Reporting of Diagnostic Accuracy (STARD) reporting guidelines.

### FLIm Analysis According to the Plaque Compositions

Data obtained from OCT-FLIm and IVUS were systematically analyzed for plaque characterization. The acquired images were examined and matched frame by frame by 2 imaging experts. Regions of interests (ROIs) were selected based on both OCT and IVUS findings following current recommendations^1,8,9,10,11^ and annotated into 5 different plaque compositions: normal arterial wall, fibrosis, superficial calcium, healed plaque, and macrophages. Thin-cap fibroatheroma (TCFA) was determined to be present when both OCT and IVUS criteria were met.^12^ A total of 1879 ROIs were analyzed—of which 455 were normal; 299 fibrosis; 153 calcium; 543 healed plaque; and 429 macrophage—with FL values extracted for each ROI across 3 distinct spectral bands. For 3-dimensional (3-D) visualization, FL-based plaque compositions were volume rendered and mapped onto 3-D–reconstructed OCT images using the OsiriX DICOM viewer (OsiriX Foundation). The eMethods section in Supplement 1 provides a detailed definition of plaque characterization, ROI sampling, and the FLIm reproducibility analysis.

To validate FLIm signatures for identifying macrophage accumulation, healed plaques, and calcification, ex vivo OCT-FLIm and histopathological examinations were performed using freshly resected human arterial specimens. These included 3 coronary arteries from heart transplant recipients, 5 aortas, 1 renal artery, and 2 inferior epigastric arteries. The overall details of ex vivo OCT-FLIm and histopathological assessment are provided in the eMethods section in Supplement 1.

### Comparison of FLIm Signature in Culprit/Target Plaques

Target/culprit plaque segments were identified based on coronary angiography and OCT findings by 2 imaging experts blinded to the FLIm and clinical information. The FLIm signature of each target/culprit segment was represented by the maximum FL values obtained from the 3 distinct spectral bands. The maximum FL value from each spectral band was determined by averaging the 10 highest FL measurements within each segment. The quantified maximum FL values in each spectral band within the target/culprit segments were compared according to the patient’s clinical presentation (ACS vs CSA).

Among the 40 patients, coronary lesion progression was assessed in 31 patients who had previously undergone coronary or cardiac computed tomographic angiography. In total, 11 rapidly progressive coronary lesions were identified in 9 patients that had not been previously observed in coronary angiographies conducted in the preceding months (mean [SD] interval between consecutive angiographies for rapid disease progression, 12.1 [4.7] months). A rapidly progressive lesion was defined based on significant changes in angiographic diameter stenosis as previously described.^13^ The FLIm signatures of the 11 progressive lesion segments were compared with those of the proximal and distal nonprogressive segments within the same vessels (intravessel analysis). Additionally, the FLIm signatures of the target/culprit lesion segments in these 9 patients with progressive CAD were compared with that of nontarget/nonculprit vessel segments in the remaining 22 patients who did not have progressive CAD, classified based on previous coronary assessments performed more than 2 years apart (intervessel analysis). The maximum FL values for each spectral band were calculated from a 5-mm-long region in the respective segments. The overall scheme and details of comparing FLIm signatures across different subsets of atherosclerotic disease activity are provided in the eMethods section and eFigure 2 in Supplement 1.

### Statistical Analysis

Continuous data were expressed as either mean (SDs) or median (IQRs), and categorical variables were expressed as frequencies (percentages). Differences in FL measurements among the 5 plaque compositions were compared using a compound symmetry covariance structure in a mixed model with a type 3 test of fixed effects. FLIm repeatability was assessed using the intraclass correlation coefficient and Bland-Altman analyses. Segmental, intervessel, and histopathological comparisons of the FLIm signatures were performed using the Wilcoxon signed rank test. Intravessel FL measurement comparisons within the target/culprit vessels were performed using the Friedman test and the Dunn post hoc pairwise comparison. The correlation between the quantitative measurements was assessed using the Spearman rank correlation test. FL differences between the 5 plaque compositions and ACS and CSA were prespecified. At the same time, analysis of target/culprit lesions with rapid angiographic progression was conducted as exploratory due to incidental identification. All statistical analyses were performed using Prism (GraphPad Software), SPSS (IBM SPSS Statistics), and R (R Foundation for Statistical Computing).

## Results

### Patient Population and Intracoronary OCT-FLIm

**Figure 1.**
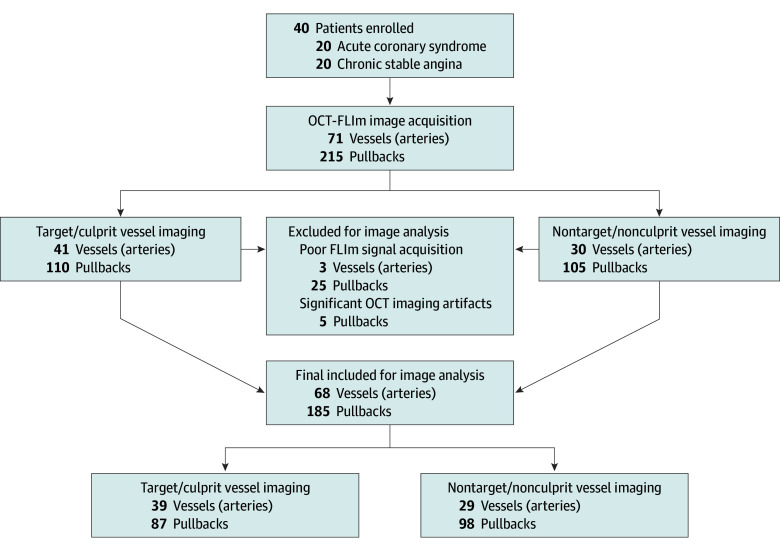
Study Flowchart of Intracoronary Optical Coherence Tomography and Fluorescence Lifetime Imaging Acquisition and Analysis FLIm indicates fluorescence lifetime imaging; OCT, optical coherence tomography.

Baseline clinical and angiographic characteristics of the patients are represented in the Table. Forty patients (mean [SD] age, 63.1 [8.1] years; 32 men [80.0%] and 8 women [20.0%]) presenting with either ACS (n = 20) or CSA (n = 20) requiring revascularization underwent preintervention OCT-FLIm scans (Figure 1). In 215 pullbacks from 71 coronary arteries, OCT-FLIm images were safely acquired during a brief, nonocclusive arterial flushing using a clinically available pentastarch solution^14^ (pullback speed, 20 mm per second; rotation speed, 100 revolutions per second; single-imaging length, 50 mm). The procedure was completed without major complications or adverse cardiac events, and there were no significant changes in renal or liver function during the 7-day follow-up period (eTable 1 in Supplement 1). A total of 185 pullback data from 68 arteries were analyzed after excluding 30 pullbacks and 3 arteries due to poor FLIm signal acquisition or significant OCT imaging artifacts (Figure 1). Consequently, of the 41 target/culprit lesions revascularized, 39 were included in the subsequent analysis.

**Table.** Baseline and Procedural Characteristics of Study Patients

	No. (%) of study population, (N = 40)
**Baseline demographic features**	
Age, mean (SD), y	63.1 (8.1)
Sex	
Male	32 (80.0)
Female	8 (20.0)
Clinical presentation	
Acute coronary syndrome	20 (50.0)
Chronic stable angina	20 (50.0)
Hypertension	24 (60.0)
Diabetes	13 (32.5)
Dyslipidemia	33 (82.5)
Renal insufficiency (eGFR of <60 mL/min)	2 (6.0)
Heart failure (LVEF of <50%)	8 (20.0)
**Laboratory findings, median (IQR)**	
Serum creatinine level, mg/dL	0.85 (0.73-1.00)
eGFR, mL/min	88.0 (71.6-105.2)
AST level, IU/L	27.5 (22.0-37.5)
ALT level, IU/L	26.5 (17.0-40.5)
Total cholesterol level, mg/dL	145.5 (120.5-185.8)
LDL-cholesterol level, mg/dL	78.0 (60.0-106.0)
hs-CRP level, mg/L	0.70 (0.28-2.05)
NT-proBNP level, pg/mL	82.7 (41.8-252.3)
HbA_1c_ level, %	5.9 (5.5-7.0)
**OCT-FLIm information**	
No. of imaged vessels	71 (100)
Left anterior descending artery	37 (52.1)
Left circumflex artery	8 (11.2)
Right coronary artery	26 (36.7)
Procedural information, median (IQR)	
Radiation dose, mGy/m^2^	509.4 (386.7-644.2)
Fluoroscopic time, min	22.8 (17.0-27.9)
Contrast volume, mL	187.5 (175.0-225.0)
Flushing volume used per pullback, mL	16.5 (17.5-21.0)

Abbreviations: ALT, alanine transaminase; AST, aspartate transaminase; eGFR, estimated glomerular filtration rate; HbA_1c_, hemoglobin A_1c_; hs-CRP, high-sensitivity C-reactive protein; LDL, low-density lipoprotein; LVEF, left ventricular ejection fraction; mGy, milligrays; NT-proBNP, N-terminal pro-B-type natriuretic peptide; OCT-FLIm, optical coherence tomographic–FL imaging.

SI conversion factors: To convert AST and ALT from IU/L to μkat/L, multiply by 0.0167; total and LDL cholesterol from mg/dL to mmol/L, multiply by 0.0259.

### FLIm Signatures According to Plaque Compositions

Based on earlier autopsy findings,^7^ the FLIm signature of each spectral band could serve as a surrogate for key pathological plaque compositions, including macrophages (inflammation-FL), loose fibrous healed tissues (healed plaque-FL), and calcium (calcification-FL). Our comparative analysis consistently showed that the presence of healed plaques lengthened the healed plaque-FL (eFigure 3A in Supplement 1). At the same time, superficial calcium resulted in a reduction of calcification-FL (eFigure 3B in Supplement 1), and macrophage infiltration was associated with a significant prolongation of inflammation-FL (eFigure 3C in Supplement 1). Multiple pairwise comparisons revealed that each composition was distinguishable from the others based on the differences in the FL values across the spectral bands (eFigure 3 D-F in Supplement 1). The mean FL obtained in vivo from fibrosis, calcium, and OCT–IVUS–defined TCFA showed close agreement with previous results reported in coronary autopsy comparisons (eTable 2 and eResults in Supplement 1).^15^ Additionally, these FL values demonstrated excellent reproducibility in a repeated pullback test of 37 pairs, with consistent FLIm signatures observed across the spectral bands (intraclass correlation coefficient, >0.98; eFigure 4 in Supplement 1).

### OCT-FLIm for the Structural-Molecular Characterization of Superficial Calcium

**Figure 2.**
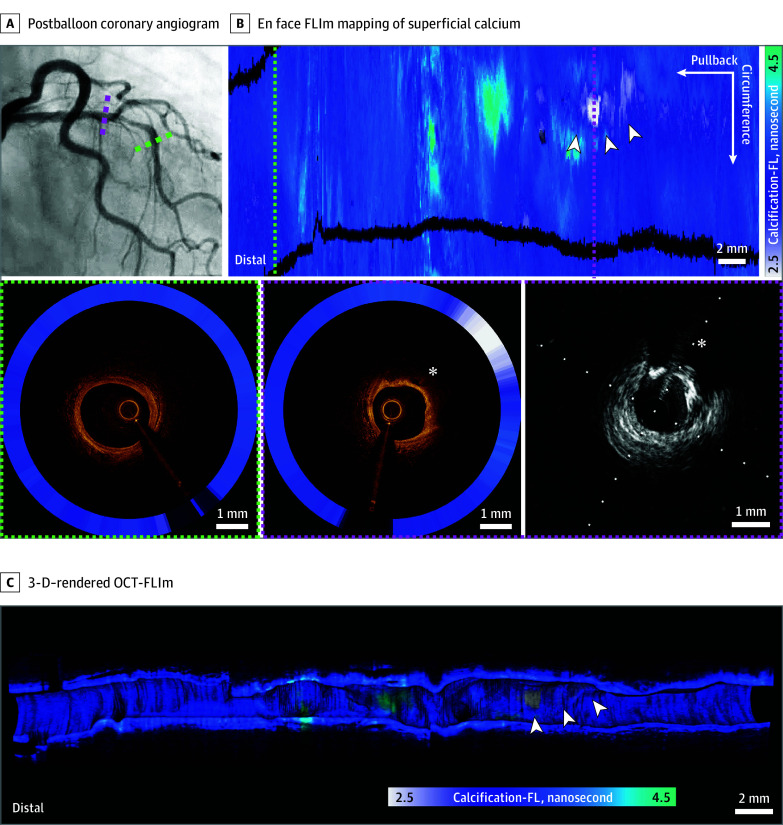
Optical Coherence Tomography and Fluorescence Lifetime Images of Superficial Calcification A, The postballoon coronary angiogram shows a calcified stenotic lesion in the proximal left anterior descending artery. B, The en face fluorescence lifetime imaging (FLIm) mapping of superficial calcium, identified by the 3 arrowheads, shows the lesion harboring various plaque compositions, including normal, fibrosis, and superficial calcium. The lower panel shows the cross-sectional optical coherence tomographic (OCT)–FLIm and intravascular ultrasound images of eccentric superficial calcification. The lower left panel shows a cross-sectional image of a normal arterial wall taken from the distal portion of image. The cross-sectional OCT-FLIm in the lower right panel shows a distinct molecular signature—defined as superficial calcification taken from area marked by the magenta dots in the upper panel. The asterisk in the lower right panel shows superficial calcification. C, Represents a 3-dimentional (3-D) rendering of the compositional signature of superficial calcium as indicated by the white arrowheads. The calcification-FL (fluorescence lifetime) ranged from 2.5 to 4.5 nanoseconds.

The OCT-FLIm scan simultaneously provided microstructural imaging and FL mapping of a coronary artery with a stenotic lesion (Figure 2A), harboring various plaque compositions, including normal, fibrosis, and superficial calcium (Figure 2B and the Video). Unlike the normal arterial wall (Figure 2C), the FLIm scan captured a distinct molecular signature from OCT-IVUS–defined superficial calcification. The FL measurements obtained from the superficial calcium were significantly shortened compared with those from normal/fibrous plaques. The mean (SD) calcification-FL for normal/fibrous plaques was 3.57 (0.140 nanoseconds vs 3.09 (0.08) nanoseconds for calcium plaques (*P* < .001; eFigure 5 in Supplement 1). The 3-D–rendered OCT-FLIm scan enabled a detailed assessment of the target plaque’s microstructural and biochemical characteristics, particularly for differentiating superficial calcium from the surrounding normal fibrosis (Figure 2C).

**Video.** Intracoronary OCT-FLIm Video of Various Plaque Composition Reflecting Clinical Disease Activity Optical coherence tomography–FL imaging (OCT-FLIm) provides real-time, simultaneous visualization of plaque microstructure and composition. FLIm signatures at different spectral wavelengths correspond to specific plaque components, including calcification, macrophage-driven inflammation, and healed plaque characteristics. This video presents OCT-FLIm pullbacks from patients with varying coronary artery disease presentations: A, Severely calcified stenotic lesion following balloon dilation, with superficial calcium exhibiting shortened calcification-FL (FL). B, Marked plaque inflammation in the culprit lesion of acute coronary syndrome, indicated by increased inflammation-FL. C, Mild plaque inflammation in the target lesion of chronic stable angina, reflected by a subtle elevation in inflammation-FL. D, Predominant healed plaque features in rapidly progressive high-grade stenosis, with a significant increase in healed plaque-FL.

Ex vivo histopathological validation of superficial calcification and fibrous plaque further supported the OCT-FLIm findings (eFigure 6A-B in Supplement 1).

### OCT-FLIm Assessment of Plaque Inflammation in ACS vs CSA

**Figure 3.**
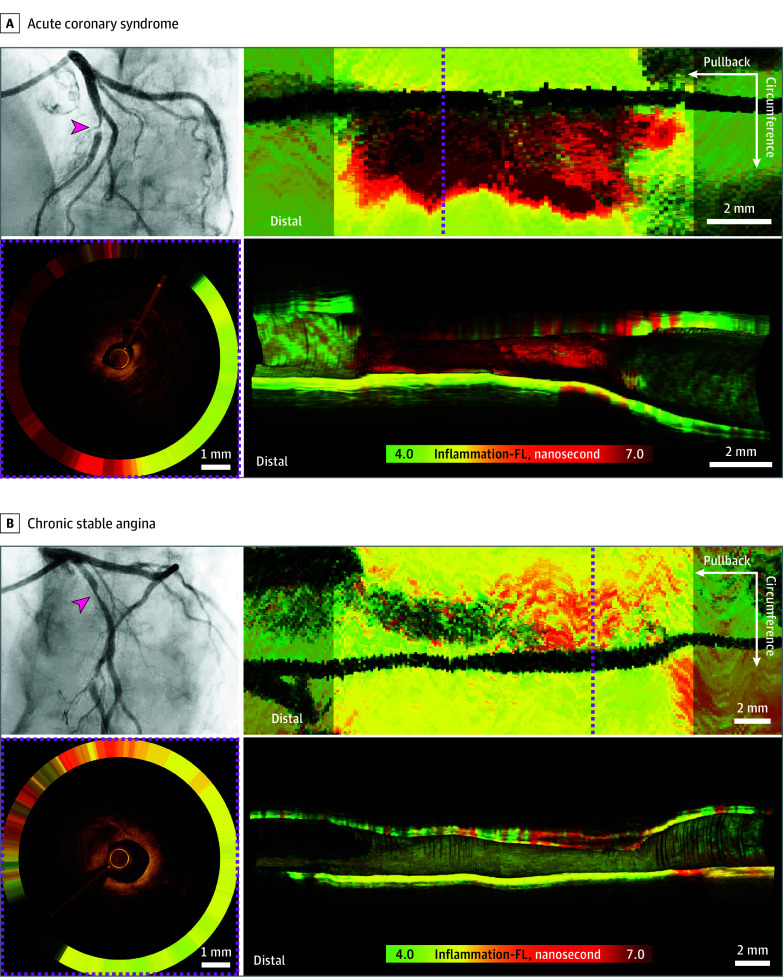
Optical Coherence Tomography and Fluorescence Lifetime Imaging Assessment of Culprit/Target Plaque Inflammation The images in A and B compare acute coronary syndrome and chronic stable angina, respectively. The upper left images show the coronary angiograms (red arrowheads, culprit/target lesions). The upper right images show the en face fluorescence lifetime imaging (FLIm) inflammation (inflammation-FL) mapping. The lower left images are cross-sectional optical coherence tomography (OCT) and FLIm scans taken from the magenta dotted lines in the upper right images. The lower right panels show the 3-dimensional rendered OCT-FLIm images. The inflammation-FL values ranged from 4.0 to 7.0 nanoseconds.

Given that inflammation promotes the transition from a stable atheroma to an unstable phenotype susceptible to acute coronary events,^2^ we compared inflammation-FL, a surrogate of plaque inflammation, at the culprit/target lesions according to the clinical presentation of the patient (ACS vs CSA; Figure 3). The current dual-modal imaging framework—offering a series of cross-sectional OCT-FLIm, en face compositional FLIm mapping, and 3-D–rendered OCT-FLIm images—characterized plaque inflammation within the imaged segments (Figure 3). OCT-FLIm visualized inflammatory activity in the culprit/target lesions of individuals presenting with ACS (Figure 3A and Video) compared with those with CSA (Figure 3B and Video). Quantitatively, of 39 target/culprit segments analyzed, the inflammatory activities in the target/culprit lesions were significantly higher in the patients with ACS than in those with CSA. The mean (SD) of inflammation-FL for those with ACS was 7.59 (0.96) nanoseconds vs 6.46 (0.87) nanoseconds for those with CSA (*P* < .001; eFigure 7 in Supplement 1).

Furthermore, ex vivo OCT-FLIm and histopathological validation confirmed the in vivo OCT-FLIm findings. The typical OCT morphology of macrophage accumulation combined with prolonged inflammation-FL (eFigure 6C in Supplement 1) was closely colocalized and correlated with histologically proven CD68-positive inflammatory cell infiltrates in the plaque regions (eFigures 6C, 8A-B, and 9A in Supplement 1).

### Comprehensive Assessment of Rapidly Progressive Lesions Using OCT-FLIm

**Figure 4.**
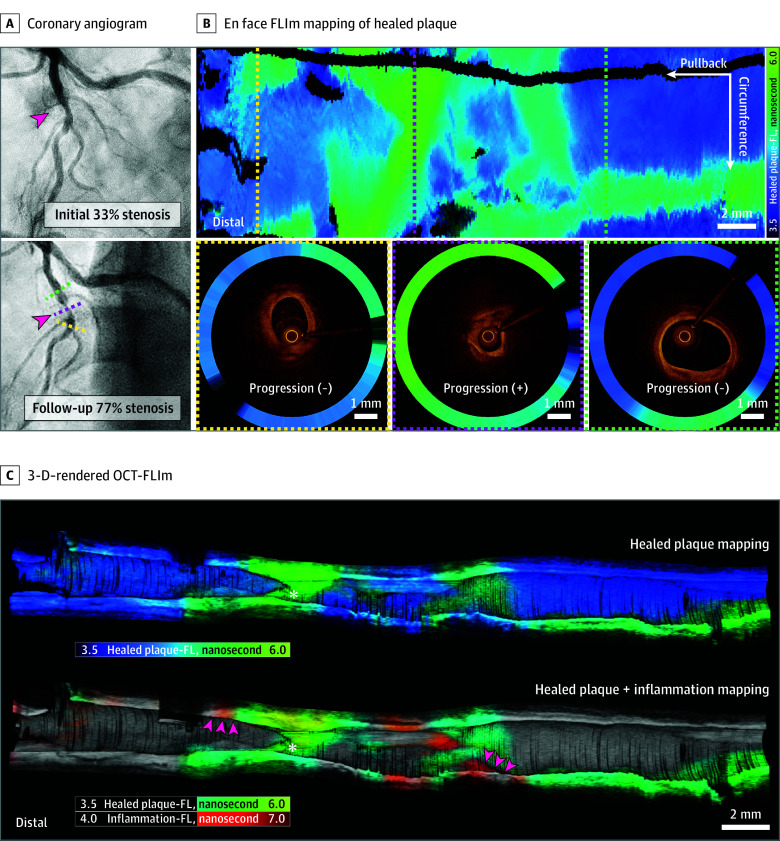
Optical Coherence Tomography and Fluorescence Lifetime Imaging Assessment of a Healed Plaque Phenotype in Rapidly Progressive Disease A, Angiographically documented rapid disease progression (red arrowheads). B, En face fluorescence lifetime imaging (FLIm) mapping of healed plaque with optical coherence (OCT)–FLIm cross-sections from distal (yellow), culprit (magenta), and proximal (green) segments. C, Three-dimensionally (3-D) rendered OCT-FLIm presenting predominant healed plaque at the culprit site (white asterisks) and closely distributed inflammatory signatures at the plaque shoulder area (red arrowheads). The healed plaque-FL and inflammation-FL values ranged from 3.5 to 6.0 nanoseconds and 4.0 to 7.0 nanoseconds, respectively.

In 11 plaque segments from 9 patients with rapidly progressive lesions showing high-grade stenosis, the baseline mean (SD) diameter of the stenosis was 31.8% (10.9%) vs follow-up progression of 80.9% (6.7%; *P* < .001; Figure 4A), FLIm accurately identified the predominant healed plaque composition, which implicated the significant role of the repetitive silent rupture and healing process in the rapid episodic progression of CAD (Figure 4B and Video). Quantitatively, using intravessel analysis, healed phenotype was more prominent in target/culprit segments than in proximal and distal nonprogressive segments. The mean (SD) of healed plaque-FL for the target/culprit segments was 5.31 (0.20) nanoseconds vs 4.65 (0.28) nanoseconds for the proximal segments (*P* < .001) and vs 4.67 (0.36) nanoseconds for distal segments (*P* = .004; eFigure 10A in Supplement 1). Likewise, the target/culprit plaques in patients with rapidly progressive CAD showed higher healed plaque-FL values than nontarget/nonculprit plaques of nonprogressive control participants. The mean (SD) healed plaque-FL was 5.31 (0.20) nanoseconds for rapidly progressive lesion segments vs 4.81 (0.30) nanoseconds for nonprogressive segments (*P* < .001; eFigure 10B in Supplement 1).

In the rapidly progressive lesions, inflammatory FLIm signatures were concurrently elevated in the target/culprit segments compared with the proximal and distal nonprogressive segments (eFigure 10C in Supplement 1). The 3-D–rendered OCT-FLIm dual imaging enabled intuitive structural-molecular characterization of the healed plaque phenotype in rapidly progressive high-grade stenosis (Figure 4C). FLIm signatures of inflammation and healed plaques were closely distributed around the plaque shoulder area in rapidly progressive lesions (Figure 4C) and correlated with each other along the target/culprit segment (*r* = 0.77, *P* < .001; eFigure 10D in Supplement 1). These data of healed plaque-FL and inflammation-FL demonstrate that multispectral detection using OCT-FLIm can identify plaques at high risk of rapid progression.

In addition, ex vivo histopathological assessments were consistent with the in vivo OCT-FLIm findings. We observed a lengthened healed plaque-FL colocalized with the layers of loosely organized type III collagen fibers (eFigures 6D, 8C and D, and 9B in Supplement 1) and identified a distinct “inflamed healed plaque” phenotype with macrophage infiltrates (eFigure 6E in Supplement 1). Furthermore, FLIm-derived compositional data complemented regular OCT or IVUS imaging to accurately assess the TCFAs and healed plaques (eFigure 11 in Supplement 1).

## Discussion

In this first-in-human diagnostic feasibility study, OCT-FLIm dual-modal intravascular imaging was demonstrated to be clinically feasible and safe for CAD assessment. FLIm enabled label-free imaging of multiple biochemical compositions in the context of the OCT-derived microstructure of coronary atheroma with high reproducibility. Ex vivo validation of human arterial segments, including freshly resected coronary arteries, strongly supported the FLIm findings in patients with CAD, showing excellent agreement with those from previous autopsy studies.^7,15^ OCT-FLIm provided real-time, structural-molecular characterization of key compositions related to high-risk plaques, including macrophages, healed plaque, superficial calcification, and fibrosis. Furthermore, heightened plaque inflammation was identified in the culprit/target lesions of ACS compared with those of CSA. Additionally, in rapidly progressive high-grade stenosis, inflammatory activity closely colocalizes with the prominent healed plaque phenotype at the target/culprit sites, suggesting a potential association between immune mechanisms and repetitive plaque rupture and healing. The current novel imaging strategy represents the first clinical application of multitargeted, nonlabeling biochemical imaging integrated with OCT morphology in patients with CAD, enabling the comprehensive characterization of coronary plaques at risk.

CAD progression involves dynamic changes in plaque composition through a complex interplay of molecular, cellular, and biochemical elements.^2,11,16,17^ Conventional intravascular imaging approaches focusing on anatomical characteristics provide limited insights into plaque pathobiology and may not fully identify plaques at risk of future coronary events.^18,19,20^ Recently, a hybrid imaging approach, the IVUS-spectroscopic lipid chemogram, has been translated into clinical practice,^20^ and has shown the superiority of a multimodal imaging strategy over stand-alone morphological information. However, the additive value of a single lipid chemogram in predicting future coronary events was somewhat less impactful than initially anticipated.^21,22^ Intravascular OCT combined with targeted near-infrared fluorescence imaging has been considered a potential alternative, enabling the molecular characterization of previously unattainable attributes of high-risk plaques, such as protease activity, inflammation, and intraplaque hemorrhages.^23,24,25,26^ However, its clinical translation remains challenging because injectable targeted contrast agents are needed. Furthermore, it remains unclear whether single-compositional imaging can sufficiently assess the complex biological features of high-risk plaques.

Considering the advantage of the inherent autofluorescence property of individual tissue constituents, FLIm can differentiate the biochemical features of atherosclerotic plaques with high sensitivity and specificity, thereby providing a valuable imaging basis for basic research.^3^ Despite its promising potential, clinical application of this approach has been impeded by major challenges, including a complex instrumental setup, slow data acquisition speed, difficulty in developing a compatible dual-modal catheter, and the need for safe blood elimination without affecting FLIm signals (eResults and eFigure 12 in Supplement 1).^14^ The integration of FLIm with OCT imaging using a dual-modal, low-profile catheter has addressed these issues, enabling simultaneous FLIm and coregistered OCT imaging at high speed in patients with CAD. Moreover, OCT-FLIm enables immediate quantitative characterization of multiple key compositions, allowing for real-time pathobiological assessment of coronary plaques.

Among the key compositions, macrophage and healed phenotype play a major role in the development of CAD, particularly by weakening the fibrous cap, which can lead to sudden rupture and rapid phasic plaque growth.^2,11,16^ Clinical trials have repeatedly demonstrated that immune modulation therapy, besides statins, such as monoclonal antibody targeting IL-1β or colchicine, can further reduce major cardiovascular events.^27,28^ Given the limited value of systemic biomarkers like C-reactive protein in improving risk stratification,^29^ clinical imaging approaches enabling locoregional assessment of coronary inflammation are now warranted to identify high-risk individuals who may benefit from immune modulation therapy.^30^ Although positron emission tomography imaging has been used to assess inflammation in large arteries,^31,32,33^ its application for coronary beds remains challenging due to low spatial resolution and interference from left ventricular fluorodeoxyglucose uptake. Contemporary OCT has been tested for identifying plaque macrophages^1,34^; however, its clinical practicality remains unclear because OCT images are highly susceptible to complex light-scattering artifacts and other components that produce bright spots.^35,36,37^

The ability of FLIm to visualize plaque macrophages has been demonstrated in preclinical studies and autopsied human atheromas.^5,6,7,15^ A recent systematic autopsy analysis revealed an association between intimal macrophage accumulation and inflammatory FLIm signals,^7^ which is consistent with our clinical OCT-FLIm study findings. This study used OCT-FLIm to visualize and quantify active inflammation in high-risk coronary plaques associated with clinical disease presentations. Inflammatory activity is significantly higher at target/culprit sites in patients with ACS than in those with CSA, reflecting the immune mechanisms underlying the plaques at risk.^2,16^ Altogether, OCT-FLIm provides key compositional indices for assessing the risk of individual plaques, with potential applications in refining immune modulation strategies for patients with ACS with a high inflammatory burden and monitoring their therapeutic response.^38^

Recent evidence suggests that a healed atheroma with a layered property contributes to rapid phasic lesion growth.^11^ The mechanism of plaque layering is a repetitive subclinical plaque rupture or erosion with the subsequent collagenous organization of mural thrombus at disrupted sites, suggesting a potential association with high-risk plaque behavior.^2,11^ Histopathologically, the healed layer is loosely organized and consists mainly of type III collagen, in contrast to the underlying fibrotic plaque enriched in type I collagen.^2,11^ The intravascular OCT-FLIm yielded a capability to image unique healed compositions by capturing FLIm signals indicative of loose fibrous tissue, complementing stand-alone OCT imaging. In rapidly progressive stenotic lesions, FLIm revealed abundant active inflammation around the predominant healed plaque phenotype at the target/culprit sites, implicating locoregional plaque instability. Given its ability to assess plaque pathobiology through multitargeted imaging, this novel OCT-FLIm system has potential as a tool for personalized CAD diagnosis and treatment strategies.

### Limitations

This study has several limitations. Although the current OCT-FLIm clinical data are strongly supported by previous autopsy studies, a systematic matching comparison with fresh coronary atheromas would be helpful for further validation, particularly for improving the accuracy of detecting extracellular lipids. Furthermore, a dedicated classification framework should be implemented to effectively manage massive amounts of FLIm data and enhance its clinical applicability. Imaging protocols should be optimized for specific clinical scenarios to minimize fluoroscopic time and contrast volume while maintaining the benefits of OCT-FLIm.

## Conclusions

Given the success of the first-in-human study, OCT-FLIm has demonstrated its feasibility as a clinically applicable, multitargeted intravascular imaging technique, enabling the simultaneous assessment of the microstructure and biochemical properties of coronary atherosclerotic plaques. Additionally, well-designed prospective multicenter clinical trials are warranted to determine whether an OCT-FLIm-guided individualized strategy can influence the clinical outcomes of patients with CAD.

## Notes

Supplement 1.
eMethods

eResults
**eTable 1.** Periprocedural renal and liver function test results **eTable 2.** Comparison of fluorescence lifetime values based on plaque tissue types **eFigure 1.** Schematic illustration of intracoronary OCT-FLIm **eFigure 2.** Comparison of lesion segments with angiographic rapid progression and non-progression **eFigure 3.** Pairwise comparison of multispectral FL values according to plaque composition **eFigure 4.** Reproducibility validation for multispectral FLIm data **eFigure 5.** Quantitative analysis of calcification-FL measurements **eFigure 6.** Histopathological validation of OCT-FLIm images in human coronary plaques **eFigure 7.** Quantitative comparison of inflammation-FL measurements **eFigure 8.** Quantitative analysis of FLIm signals and histopathological stained areas **eFigure 9**. Histopathological validation of OCT-FLIm images in varying human arterial specimens **eFigure 10.** Quantitative analysis of FLIm signals in target/culprit lesions of rapid disease progression **eFigure 11.** OCT-FLIm images of thin-cap fibroatheroma-like lesions and atypical healed plaque morphologies **eFigure 12.** OCT-FLIm images obtained using different solutions: saline vs. pentastarch
eReferences

Supplement 2.
Data Sharing Statement
