# Supplementary material for: Intracoronary Structural-Molecular Imaging for Multitargeted Characterization of High-Risk Plaque: First-in-Human OCT-FLIm
Source: JAMA Cardiol. 2025 May 7;10(7):708–17. doi: 10.1001/jamacardio.2025.0928 (PMC12242695; doi:10.1001/jamacardio.2025.0928)
Supplement: Supplement 1. — eMethods eResults eTable 1. Periprocedural renal and liver function test results eTable 2. Comparison of fluorescence lifetime values based on plaque tissue types eFigure 1. Schematic illustration of intracoronary OCT-FLIm eFigure 2. Comparison of lesion segments with angiographic rapid progression and non-progression eFigure 3. Pairwise comparison of multispectral FL values according to plaque composition eFigure 4. Reproducibility validation for multispectral FLIm data eFigure 5. Quantitative analysis of calcification-FL measurements eFigure 6. Histopathological validation of OCT-FLIm images in human coronary plaques eFigure 7. Quantitative comparison of inflammation-FL measurements eFigure 8. Quantitative analysis of FLIm signals and histopathological stained areas eFigure 9. Histopathological validation of OCT-FLIm images in varying human arterial specimens eFigure 10. Quantitative analysis of FLIm signals in target/culprit lesions of rapid disease progression eFigure 11. OCT-FLIm images of thin-cap fibroatheroma-like lesions and atypical healed plaque morphologies eFigure 12. OCT-FLIm images obtained using different solutions: saline vs. pentastarch eReferences [file jamacardiol-e250928-s001.pdf]

Supplemental Online Content

Kim S, Nam HS, Kang DO, et al. Intracoronary structural-molecular imaging for multi-targeted characterization of high-risk plaque. *JAMA Cardiology*. doi:10.1001/jamacardio.2025.0928

eMethods

eResults

eTable 1. Periprocedural renal and liver function test results

eTable 2. Comparison of fluorescence lifetime values based on plaque tissue types

eFigure 1. Schematic illustration of intracoronary OCT-FLIm

eFigure 2. Comparison of lesion segments with angiographic rapid progression and non-progression

eFigure 3. Pairwise comparison of multispectral FL values according to plaque composition

eFigure 4. Reproducibility validation for multispectral FLIm data

eFigure 5. Quantitative analysis of calcification-FL measurements

eFigure 6. Histopathological validation of OCT-FLIm images in human coronary plaques

eFigure 7. Quantitative comparison of inflammation-FL measurements

eFigure 8. Quantitative analysis of FLIm signals and histopathological stained areas

eFigure 9. Histopathological validation of OCT-FLIm images in varying human arterial specimens

eFigure 10. Quantitative analysis of FLIm signals in target/culprit lesions of rapid disease progression

eFigure 11. OCT-FLIm images of thin-cap fibroatheroma-like lesions and atypical healed plaque morphologies

eFigure 12. OCT-FLIm images obtained using different solutions: saline vs. pentastarch

eReferences

© 2025 American Medical Association. All rights reserved, including those for text and data mining, AI training, and similar technologies.

Table of Contents

eMethods -- 3

OCT-FLIm system and dual-modal catheters -- 3

Definition of plaque characterization and ROI sampling by OCT and IVUS ----------------------- 3

Reproducibility of FLIm --- 4

Ex vivo OCT-FLIm and histopathologic evaluation of arterial segments ----------------------------- 4

Quantitative analysis of FLIm signals and immunohistochemical stained areas --------------------- 5

Definition of angiographic rapid disease progression and non-progression ------------------------- 5

Sample size determination -- 6

Definitions of safety and feasibility of intracoronary OCT-FLIm -------------------------------------- 6

eResults -- ------------- 7

Composition-wise FL spectra in comparison with previous data -------------------------------------- 7

Safe blood elimination without affecting FLIm signal --- 7

eTables --- 8

eTable 1: Periprocedural renal and liver function test results --- 8

eTable 2: Comparison of fluorescence lifetime values based on plaque tissue types ---------------- 9

eFigures -- 10

eFigure 1: Schematic illustration of intracoronary OCT-FLIm -- 10

eFigure 2: Comparison of lesion segments with angiographic rapid progression and nonprogression -11

eFigure 3: Pairwise comparison of multispectral FL values according to plaque composition ---- --12

eFigure 4: Reproducibility validation for multispectral FLIm data ------------------------------------- 13

eFigure 5: Quantitative analysis of calcification-FL measurements ------------------------------------- 14

eFigure 6: Histopathological validation of OCT-FLIm images in human coronary plaques ------- 15

eFigure 7: Quantitative comparison of inflammation-FL measurements ------------------------------ 16

eFigure 8: Quantitative analysis of FLIm signals and histopathological stained areas --------------- 17

eFigure 9: Histopathological validation of OCT-FLIm images in varying human arterial specimens - 18

eFigure 10: Quantitative analysis of FLIm signals in target/culprit lesions of rapid disease progression --19

eFigure 11: OCT-FLIm images of thin-cap fibroatheroma-like lesions and atypical healed plaque morphologies--- 20

eFigure 12: OCT-FLIm images obtained using different solutions: saline vs. pentastarch --------- 21

eReferences -- 22

© 2025 American Medical Association. All rights reserved, including those for text and data mining, AI training, and similar technologies.

OCT-FLIm system and dual-modal catheters

We developed a fully integrated, high-speed optical coherence tomography-fluorescence lifetime imaging (OCT-FLIm) system for clinical application.[1] The custom-built, dedicated hybrid optical rotary joint enabled a high-throughput optical combination of OCT and FLIm while rotating at a high speed up to 100 rps. A 2.6-French low-profile, dual-modality imaging catheter based on a double-clad fiber (DCF13; Thorlabs Inc., NJ, USA) was devised to deliver and receive combined OCT and FLIm excitation/emission lights effectively. The optical power for OCT imaging was approximately 18 mW at the catheter tip. The 355 nm ultraviolet radiation energy of this system was only 0.35 mJ/cm^2, which is far below the maximum permissible exposure limit for the skin (3.52 mJ/cm^2) in accordance with laser safety guidelines (American National Standard for Safe Use of Lasers in Health Care; ANSI Z136.3).[2]

Autofluorescence signals from coronary plaques were spectrally separated into three distinct bands: 390/40 nm, 450/45 nm, and 540/50 nm (center wavelength/bandwidth). This approach ensured effective measurement of fluorescence lifetime (FL) across multiple spectral bands while minimizing potential issue of crosstalk among multispectral FLIm signals. The FL information was then reconstructed instantaneously from the raw signals using the high-speed analog mean-delay method.[3] To improve clarity and facilitate interpretation of multispectral FL measurements, each spectral band was designated with clinically relevant terms based on its predominant compositional targets: 390 nm band as 'healed plaque-band', 450 nm band as 'calcification-band', and 540 nm band as 'inflammation-band'. The corresponding FL values were subsequently labeled as 'healed plaque-FL', 'calcification-FL', and 'inflammation-FL', respectively.

The FLIm system operated in synchronization with a swept-source engine (AXP50124-17, Excelitas Technologies Corp., MA, USA) at a sweeping rate of 100 kHz, thus yielding multispectral FL measurements in every four OCT A-lines (FL measurement rate of 25 kHz). The acquired data from OCT and FLIm were parallel-processed, enabling real-time visualization of co-registered OCT-FLIm images. The entire procedure, including real-time image acquisition, visualization, and plaque characterization, was implemented using a dedicated user interface written in C++. The system was tested and found to comply with the International Electrotechnical Commission standards of the Korea Testing Laboratory and Korea Ministry of Food and Drug Safety.

Definition of plaque characterization and ROI sampling by OCT and IVUS

Region of interests (ROIs) were selected based on both OCT and intravascular ultrasound (IVUS) findings and annotated into five different plaque compositions: normal arterial wall, fibrosis, superficial calcium, healed plaque, and macrophages, with an agreement of two imaging experts (E.J.P. and D.O.K.). Arterial walls showing a well-delineated three-layered architecture comprising thin intima, media, and adventitia were considered normal. Fibrosis was defined as a plaque area exhibiting a homogeneous, high-backscattering signal on OCT without definite hypoechoic areas on the corresponding IVUS images.[4,5]

© 2025 American Medical Association. All rights reserved, including those for text and data mining, AI training, and similar technologies.

Superficial calcium was determined to be a signal-poor or heterogeneous region with a sharply delineated border on OCT and brightly echogenic foci with acoustic shadowing on the corresponding IVUS.[4,5] Healed plaque was defined as a superficial tissue layer of different optical intensity that was demarcated from the underlying plaque on OCT.[6-8] We defined macrophage infiltrates as signal-rich, distinct, or confluent punctate areas with shadowing on OCT.[4] Thin-cap fibroatheroma (TCFA) was determined when both OCT and IVUS criteria were met, as previously suggested.[9] OCT-derived TCFA was defined as a plaque with a lipid arc of > 90 degree and a fibrous cap of < 65 μm in at least three consecutive frames. IVUS-derived TCFA was described as a lesion that met the following criteria: > 180 degree of echo attenuated area without calcium or > 90 degree of lipid pool-like images in at least five consecutive frames and plaque burden > 50%.[9] Frames that did not meet the specified criteria or contained significant artifacts were excluded. To avoid repeated measurements and ensure spatial independence, only one pullback per vessel was analyzed, with each sampling was restricted to a single ROI within a 1 mm segment. Theoretically, each patient could contribute a maximum of three pullbacks from each coronary vessel (left anterior descending, left circumflex arteries, and right coronary artery), allowing for up to a maximum of 150 ROIs per patient.

Reproducibility of FLIm

To test the reproducibility of FLIm signatures, we compared the FL signals of each spectral band across two consecutive imaging pullbacks obtained from an identical segment. Among the 41 pairs of repeated pullbacks, 37 pairs were finally analyzed after excluding four sets of imaging data with poor FLIm signal acquisition and significant OCT imaging artifacts. Repeatability was assessed using the intraclass coefficient and Bland–Altman analyses.

***Ex vivo* OCT-FLIm and histopathologic evaluation of arterial segments**

Fresh specimens of three coronary arteries, five aortas, one renal artery, and two inferior epigastric arteries kept in phosphate-buffered saline were imaged using OCT-FLIm immediately after surgical resection (within four hours post-excision). Coronary arteries were obtained from heart transplantation recipients and imaged intact as part of the whole heart. Non-coronary arterial segments were imaged mounted in a custom-made wire holder after tissue preparation. After imaging, the arteries embedded in optimal cutting temperature compound were frozen and serially sectioned on a cryostat-microtome at 10 μm thickness. We used CD68 macrophage-specific monoclonal antibody (M0876; Dako, Glostrup, Denmark) to label plaque macrophages. Sections stained with picrosirius red (PSR; ScyTek Inc., Logan, UT, USA) were evaluated under a polarized light microscope to identify loosely organized type III collagen fibers.[8] Von Kossa staining (ScyTek Inc., Logan, UT, USA) was used for detecting calcium deposits within the plaques and vessel walls. The collection and use of human arterial specimens for this study was approved by the Institutional Review Board of Korea University Guro Hospital (2022GR0086).

© 2025 American Medical Association. All rights reserved, including those for text and data mining, AI training, and similar technologies.

Quantitative analysis of FLIm signals and immunohistochemical stained areas

Ex vivo OCT-FLIm images of coronary arteries were precisely matched with histopathological sections using reproducible landmarks such as side branches and calcium deposits. To ensure accurate quantitative analysis, paired OCT-FLIm images and histological sections were co-registered via affine transformation of the corresponding histological images. Following co-registration, each paired image was divided azimuthally into 30-degree sectors based on the lumen center. For CD68 immunochemistry, quantitative analysis of positive staining was conducted using the ImmunoHistoChemistry (IHC) profiler[10], an objective and unbiased automated digital immunohistochemistry image analysis software. The IHC profiler quantitatively evaluates regions with positive staining from a specific antibody, scaling the staining intensity from zero to one. Using this tool, we extracted images that reflect the intensity of antibody staining. For PSR polarized images, we developed a custom analysis tool to differentiate green-colored regions, indicative of type III collagen fibers[11]. This method enabled accurate estimation of the quantitative staining intensity of type III collagen in PSR polarized images. After applying the respective quantitation methods to the corresponding histological images, we calculated the positive staining area for each 30-degree sector by dividing the number of positively stained pixels by the total number of pixels in the sector area. The radial length of each sector was limited up to 200 μm, considering the penetration depth of ultraviolet excitation light. Finally, the averaged FLIm signals of each spectral band were compared with the co-registered, quantified sectioned areas to establish the correlation between FLIm signals and plaque compositions, such as macrophages and loose fibrous tissue. For further comparative analysis, optimal diagnostic threshold lifetime values for macrophages and loose fibrous tissue were determined, presenting the best sensitivity and specificity through ROC analysis (data not shown). We then compared the FL distributions between regions positive and negative for macrophages or loose fibrous tissue to elucidate these relationships. Spearman's rank correlation test assessed correlations between multispectral FLIm signals and histopathological stained areas. Histopathological comparisons of FLIm signature employed Wilcoxon signed-rank test. These quantitation processes were implemented using ImageJ (National Institute of Health, Bethesda, MD, USA) and MATLAB (R2022a; The MathWorks, Natick, MA, USA).

Definition of angiographic rapid disease progression and non-progression

In our study, there were nine patients (11 lesions) presenting with rapidly progressive coronary artery disease (CAD), as documented by serial angiography during clinical follow-up before enrollment. Rapidly progressive lesion was defined as a lesion showing: (1) > 10% diameter reduction of a pre-existing stenosis > 50%, (2) > 30% diameter reduction of a pre-existing stenosis < 50%, (3) progression of any lesion to total occlusion at the second angiogram, (4) 'new' lesions were a diameter reduction > 30% in a segment that was normal at the first angiogram.[12] The interval between the consecutive angiograms was set at a maximum of 18 months. Among the remaining 31 patients, 22 were classified as having non-progressive CAD at the non-target/non-culprit vessel segments based on screening coronary or cardiac computed

© 2025 American Medical Association. All rights reserved, including those for text and data mining, AI training, and similar technologies.

tomography angiography conduced more than two years before study enrollment, confirming stable or non-progressing CAD during that period. eFigure 2 shows the overall scheme comparing lesion segments with angiographic rapid progression and non-progression.

Sample size determination

This first-in-human feasibility trial aims to validate the efficacy and clinical applicability of a novel OCT-FLIm technology, as well as to assess the FLIm capability to differentiate high-risk plaques from stable ones. This study planned to enroll a total of 40 patients based on the following rationale. Since there is a lack of clinical data reporting FL measurements acquired *in vivo* from human coronary atherosclerotic plaques, the sample size was determined by extrapolating from the results of previous autopsy studies.[13] Considering the previously reported average FL values of TCFAs (390 nm band FL: 3.44 ± 0.44 ns) and fibrocalcific plaques (390 nm band FL: 3.96 ± 0.39 ns),[13] and aiming for a two-sided test with a p-value of .05 and a statistical power of 95%, a minimum of 18 plaques per group (acute coronary syndrome vs. chronic stable angina) is required to demonstrate a significant difference in FL between them. Assuming a dropout rate of approximately 10%, a total of 40 participants were planned for recruitment.

Definitions of safety and feasibility of intracoronary OCT-FLIm

This study recorded significant OCT-FLIm-related complications judged by operator that occurred during or within 24 hours of an OCT-FLIm examination.[14] Major complications were defined as significant arrhythmia, embolization, and coronary dissection, perforation or spasm that occurred in association with OCT-FLIm acquisition. Adverse cardiac events were defined as myocardial infarction, emergent unplanned revascularization, and any death related to OCT-FLIm acquisition procedure. All risks were classified by outcome as immediate correction (no action required), transient with treatment before leaving the catheterization laboratory, or requiring treatment/surveillance after leaving the catheterization laboratory, including lengthening patient's hospitalization. Feasibility of intracoronary OCT-FLIm was defined as the capability to obtain a good quality of combined OCT and FLIm images simultaneously without significant artifacts, as judged by operator and FLIm technician. Periprocedural safety parameters of intracoronary OCT-FLIm included the total radiation dose and contrast volume administered during the procedure, average flushing volume used per pullback, as well as renal and liver functional tests.

© 2025 American Medical Association. All rights reserved, including those for text and data mining, AI training, and similar technologies.

eResults

Composition-wise FL spectra in comparison with previous data

To evaluate whether the present FLIm findings were corroborated by previous data, we searched the literature and found one study reporting the detailed spectral-wise statistics of FL obtained from autopsied coronary arteries.[13] Specifically, the study reported a mean 390 nm band FL value of 4.19 ± 0.29 nsec in fibrotic plaques. Fibrocalcific lesions had lower 390 nm band FL values (3.96 ± 0.39 nsec) than fibrotic tissues. A significant decrease in 390 nm band FL (3.44 ± 0.44 nsec) was reported in TCFA, which was primarily attributable to high lipid and macrophage infiltration and the resultant collagen depletion.[13] Our FLIm findings not only showed similar trends in composition-wise FL variation but also had good agreement of the mean FL values with those from autopsied plaques.[13] The mean FL values of the same spectral band obtained *in vivo* in our study and those obtained from autopsied plaques of a corresponding histopathologic stage are presented in eTable 2.[13] Although statistical comparisons between the two sets of FL measurements were unavailable, the composition-wise mean FL values were very similar between them. Some marginal differences in FL statistics might be attributed to different measurement environments.

Safe blood elimination without affecting FLIm signal

An aqueous medium that facilitates complete blood elimination without undermining ultraviolet light transmission or affecting the original fluorescence emission is essential in obtaining intracoronary FLIm signal. Although normal saline (0.9% NaCl) is highly feasible for FLIm acquisition,[15] its low viscosity may impede effective blood clearing. We evaluated the feasibility of pentastarch (Jeil Pentastarch 10%, Jeil Pharmacy, South Korea), a clinically available solution with higher viscosity also usable for OCT imaging,[16] for obtaining FLIm in comparison with the normal saline. A freshly resected human inferior epigastric artery was repeatedly imaged in a custom-made *ex vivo* imaging chamber filled differently with the normal saline and the pentastarch. FLIm parameters averaged per frame were compared between the two conditions. As shown in eFigure 12, FL images obtained using the two respective media showed close homogeneity without signs of significant optical interference, indicating that pentastarch solution has high optical transparency for ultraviolet and visible lights at comparable level to those of normal saline. There were no differences with respect to the averaged multispectral FL measurements between the two repeated pullbacks (intraclass correlation coefficients: healed plaque-FL, 0.90, $P < .001$; calcification-FL, 0.98, $P < .001$; inflammation-FL, 0.91, $P < .001$; eFigure 12C, F, and I).

© 2025 American Medical Association. All rights reserved, including those for text and data mining, AI training, and similar technologies.

eTables

eTable 1. Periprocedural renal and liver function test results after imaging

	Baseline	7-day follow-up	P-value
**Renal function test**			
Creatinine level (mg/dL)	0.85 (0.73–1.00)	0.83 (0.66–0.93)	.13
Cystatin C level (mg/dL)	0.90 (0.79–1.15)	0.95 (0.80–1.10)	.59
eGFR (mL/min)	88.0 (71.6–105.2)	92.8 (78.3–103.8)	.12
**Liver function test**			
AST level (IU/L)	27.5 (22.0–37.5)	33.0 (23.0–42.8)	.25
ALT level (IU/L)	26.5 (17.0–40.5)	33.0 (23.0–42.8)	.12
Total bilirubin level (mg/dL)	0.57 (0.46–0.77)	0.57 (0.44–0.76)	.89

Data are expressed as medians (interquartile ranges). ALT, alanine transaminase; AST, aspartate transaminase; eGFR, estimated glomerular filtration rate.

© 2025 American Medical Association. All rights reserved, including those for text and data mining, AI training, and similar technologies.

eTable 2. Comparison of fluorescence lifetime values based on plaque tissue types

Composition/histopathology	Spectral band (nm)	FL (*in vivo* live plaque; nsec)	FL (*ex vivo* autopsied plaque; nsec)[13]
Fibrous/fibrotic	390	4.11 ± 0.25	4.19 ± 0.29
Calcium/fibrocalcific	390	3.98 ± 0.29	3.98 ± 0.39
Thin-cap fibroatheroma	390	3.20 ± 0.44	3.44 ± 0.44

Data are expressed as mean ± standard deviation, with mean FL values presented for each plaque subtype. Histopathological classification is defined as follows:[13] fibrotic = tissue regions in a stable fibrous cap that is predominantly composed of densely packed mature collagen; fibrocalcific = fibroatheroma with a cap that is predominantly calcified with high collagen content; Thin cap fibroatheroma = fibroatheroma with a thin cap (< 65 μm) rich in macrophages/lymphocytes and sparse collagen and smooth muscle cells. FL, fluorescence lifetime.

© 2025 American Medical Association. All rights reserved, including those for text and data mining, AI training, and similar technologies.

eFigures

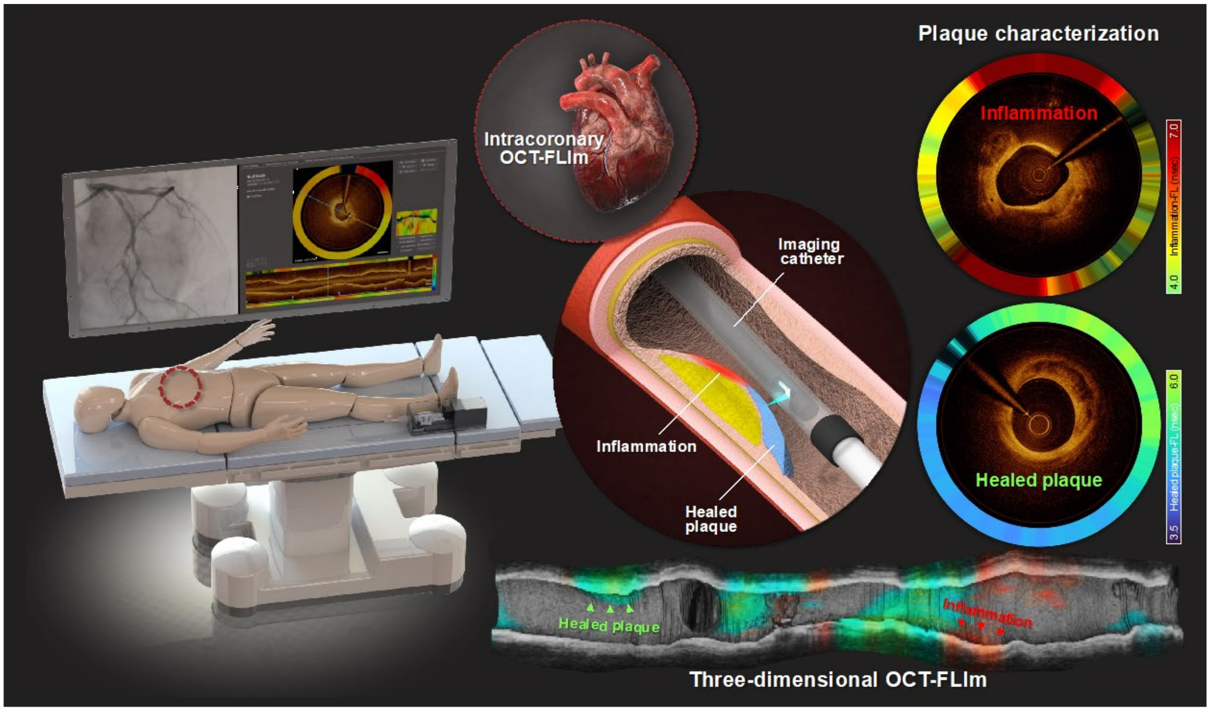

eFigure 1. Schematic illustration of intracoronary OCT-FLIm

Schematic illustration of intracoronary OCT-FLIm enabling comprehensive structural-molecular imaging of coronary plaques. FL, fluorescence lifetime.

© 2025 American Medical Association. All rights reserved, including those for text and data mining, AI training, and similar technologies.

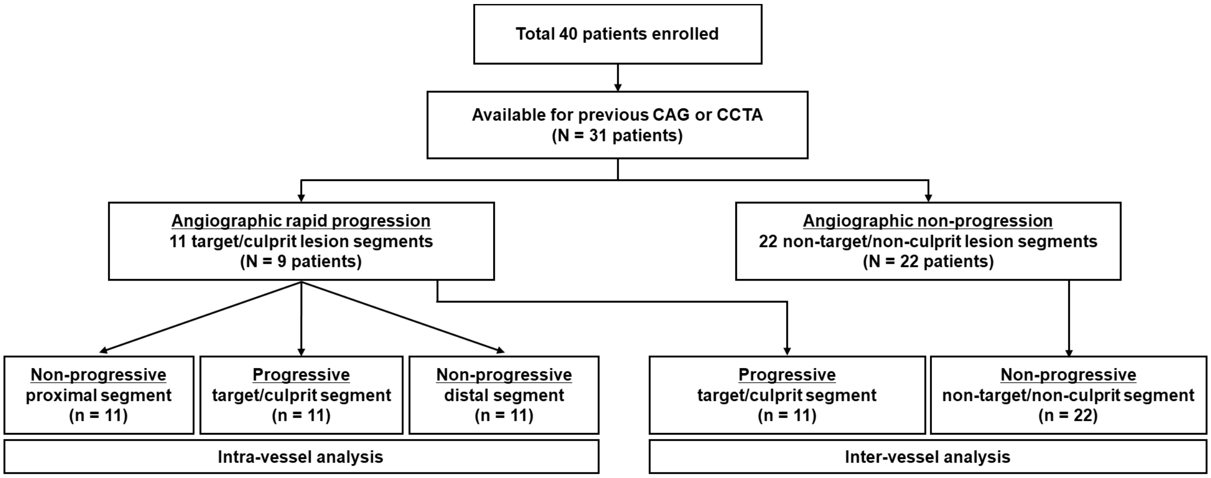

eFigure 2. Comparison of lesion segments with angiographic rapid progression and non-progression

The diagram outlines the number of patients and lesion segments categorized into two groups: angiographic rapid progression and non-progression. Angiographic rapid progression was defined based on a pre-specified criteria assessed over a maximum of 18 months. Non-progression was confirmed through coronary imaging assessment (CAG or CCTA) conducted over a minimum of two years. CAG, coronary angiography; CCTA, coronary computed tomography angiography.

© 2025 American Medical Association. All rights reserved, including those for text and data mining, AI training, and similar technologies.

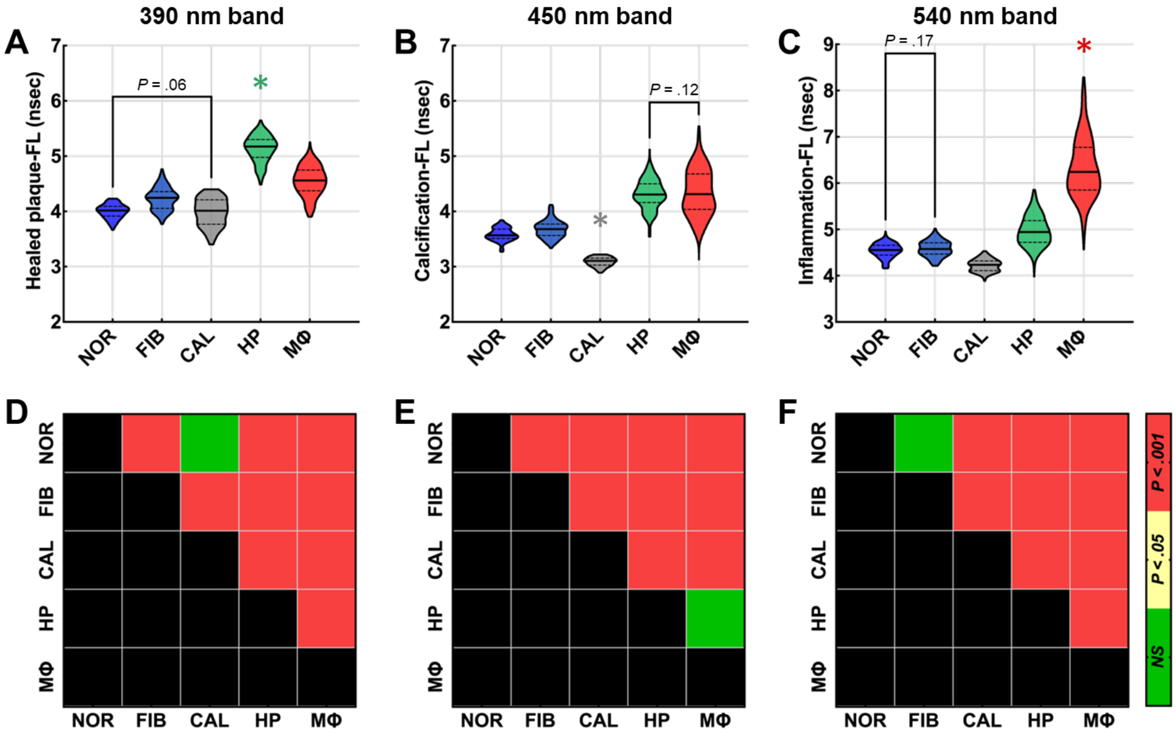

eFigure 3. Pairwise comparison of multispectral FL values according to plaque composition

(A-C) Quantitative analysis of fluorescence lifetime measurements across different spectral bands for various plaque compositions. Solid center lines and dashed lines of violin plots represent the medians and quartile ranges, respectively. All pairwise comparisons were statistically significant (P < .001) unless otherwise specified. Healed plaque-, calcification-, and inflammation-FLs correspond to fluorescence lifetimes at spectral bands centered at 390 nm, 450 nm, and 540 nm, respectively. Colored asterisks indicate the representative plaque compositions distinguishable by each spectral band. **(D-F)** Confusion matrix assessing statistical significance in each spectral band. Heat maps show the significances (p-value) of the multiple pairwise comparison results as a grid of colored squares. FL, fluorescence lifetime; NOR, normal; FIB, fibrosis; CAL, calcium; HP, healed plaque; MΦ, macrophages; NS, non-significant (P > .05).

© 2025 American Medical Association. All rights reserved, including those for text and data mining, AI training, and similar technologies.

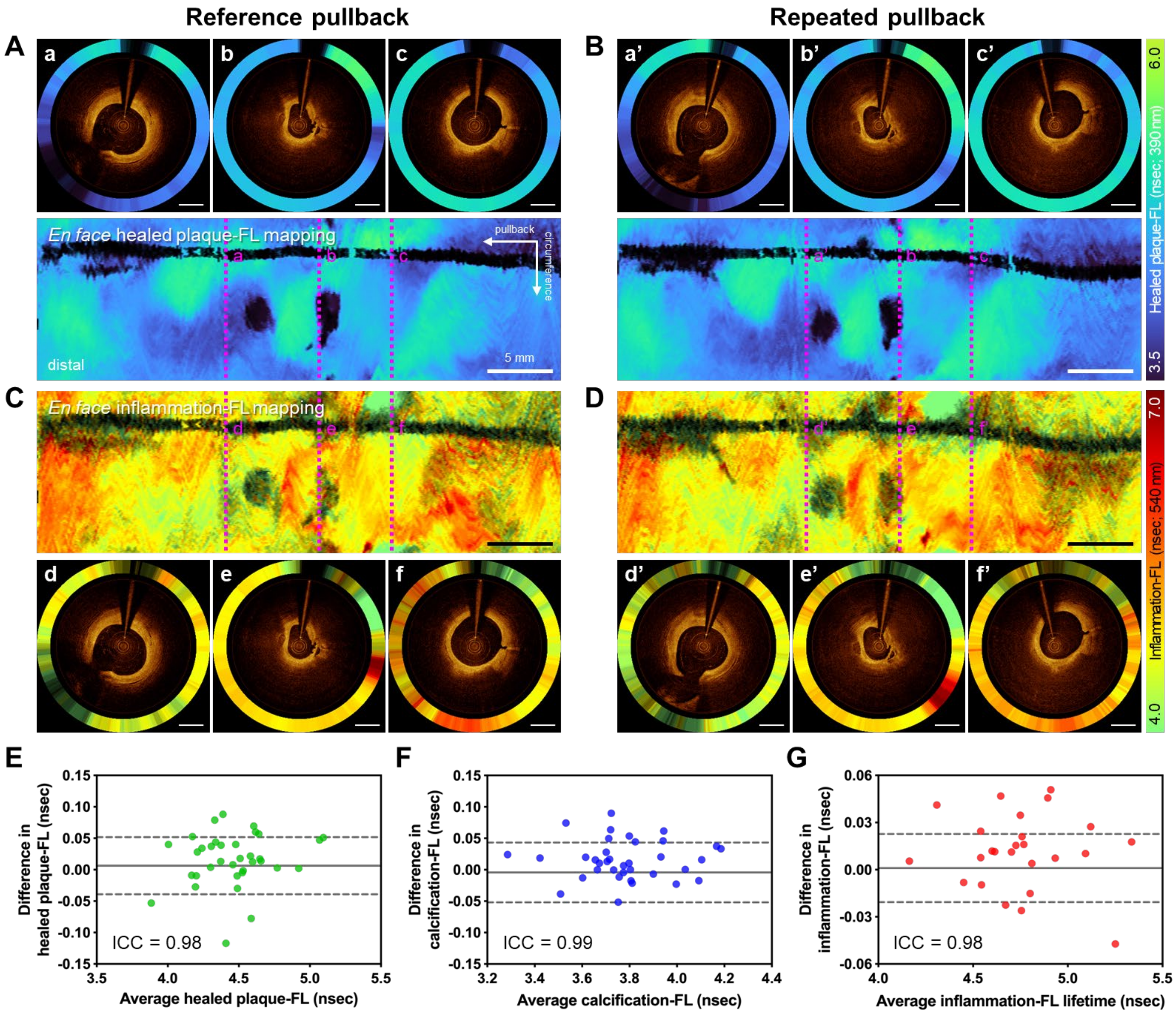

eFigure 4. Reproducibility validation for multispectral FLIm data

(A-D) Cross-sectional and *en face* images demonstrate close reproducibility across repeated pullbacks for healed plaque- (A-B) and inflammation- (C-D) fluorescence lifetime (FL). **(E-G)** Bland–Altman analysis for healed plaque- (E), calcification- (F), and inflammation- (G) FL: differences between the repeated imaging datasets were plotted against their means and 95% agreement limit. FL, fluorescence lifetime; ICC, intraclass correlation coefficient.

© 2025 American Medical Association. All rights reserved, including those for text and data mining, AI training, and similar technologies.

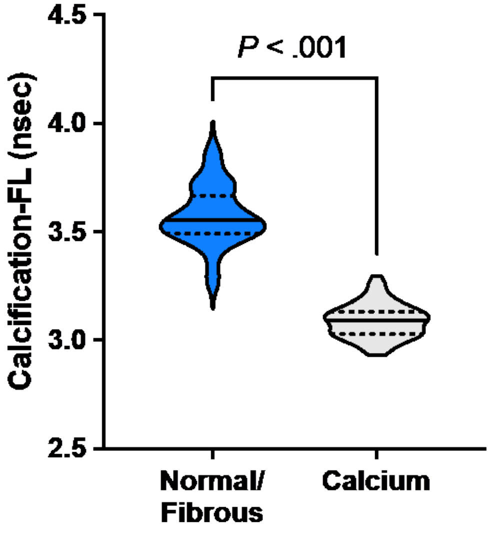

eFigure 5. Quantitative comparison of calcification-FL measurements

Quantitative analysis of calcification-FL measurements comparing FL values between normal/fibrotic tissue and calcium deposits. Calcified regions show shorter FL values (blue distribution), while normal and fibrous regions present significantly longer FL values (white distribution). FL, fluorescence lifetime.

© 2025 American Medical Association. All rights reserved, including those for text and data mining, AI training, and similar technologies.

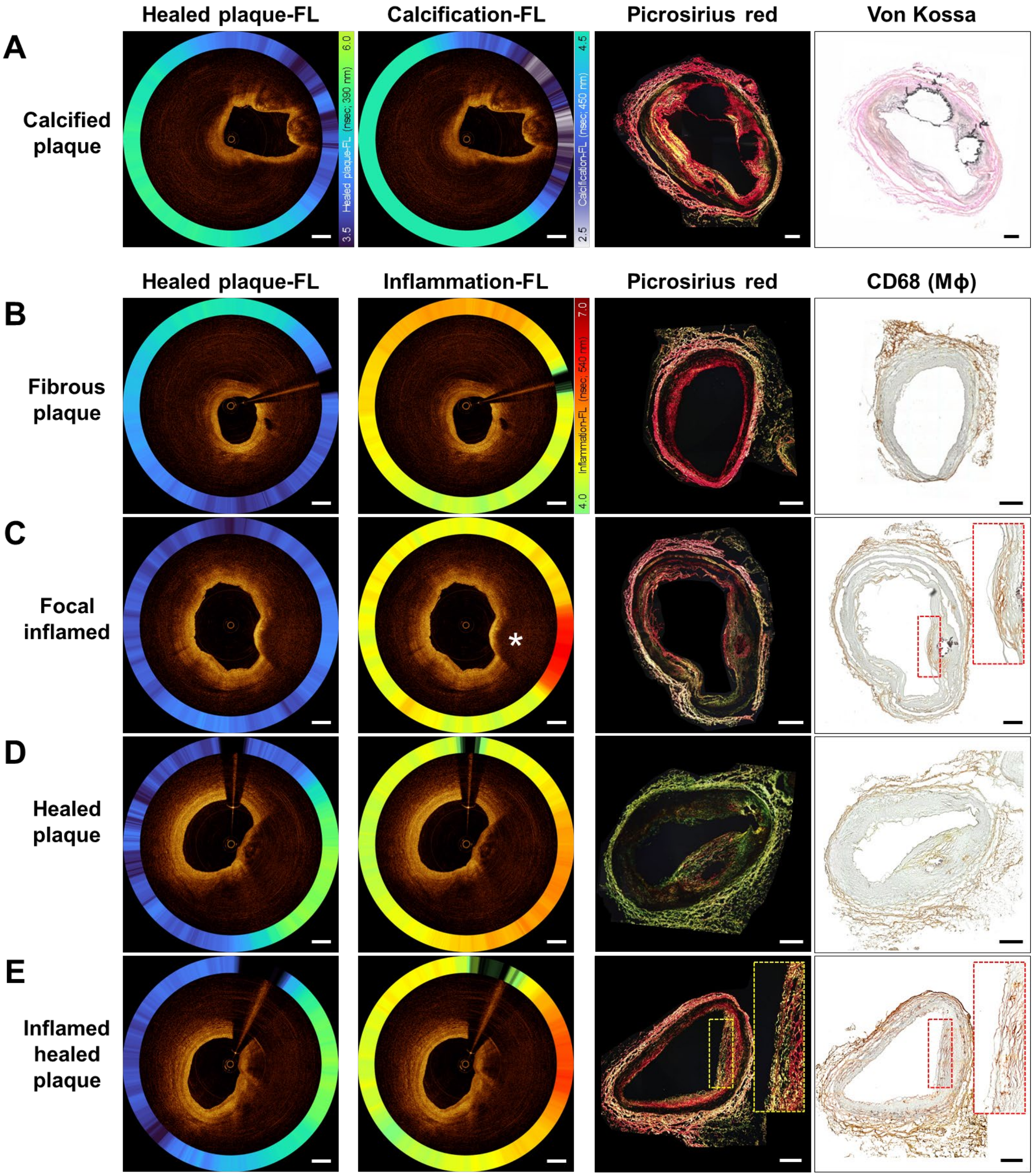

eFigure 6. Histopathological validation of OCT-FLIm images in human coronary plaques

Representative *ex vivo* OCT-FLIm and corresponding histopathologic images of various plaque subtypes obtained from freshly resected human coronary arteries are presented. **(A)** Superficial calcified nodules as indicated by Von Kossa positive areas. **(B)** Fibrous plaque. **(C)** Focal inflammation co-localized with CD68 positive macrophage infiltrates. A region with prolonged inflammation-FL in OCT-FLIm (white asterisk) corresponds to an area with CD-68 positive macrophage accumulation. **(D)** Healed plaque co-localized with loose fibrous type III collagen fibers in picrosirius red under polarized microscopy. **(E)** Inflamed healed plaque with co-existing macrophage infiltrates and loose fibrous type III collagen fibers. Scale bars indicate 1 mm for cross-sectional images. FL, fluorescence lifetime; MΦ, macrophages.

© 2025 American Medical Association. All rights reserved, including those for text and data mining, AI training, and similar technologies.

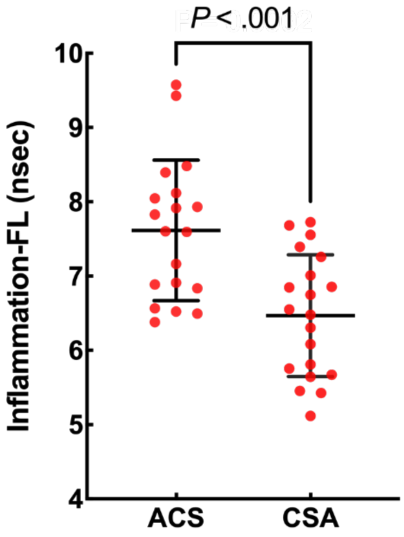

eFigure 7. Quantitative comparison of inflammation-FL measurements

Quantitative comparison of inflammation-FL measurements between culprit lesions in acute coronary syndrome (ACS) and target lesions in chronic stable angina (CSA). Analysis of 39 target/culprit segments revealed significantly higher inflammatory activity in ACS compared to CSA (inflammation-FL: 7.59 ± 0.96 vs. 6.46 ± 0.87 nsec, $P < .001$). FL, fluorescence lifetime.

© 2025 American Medical Association. All rights reserved, including those for text and data mining, AI training, and similar technologies.

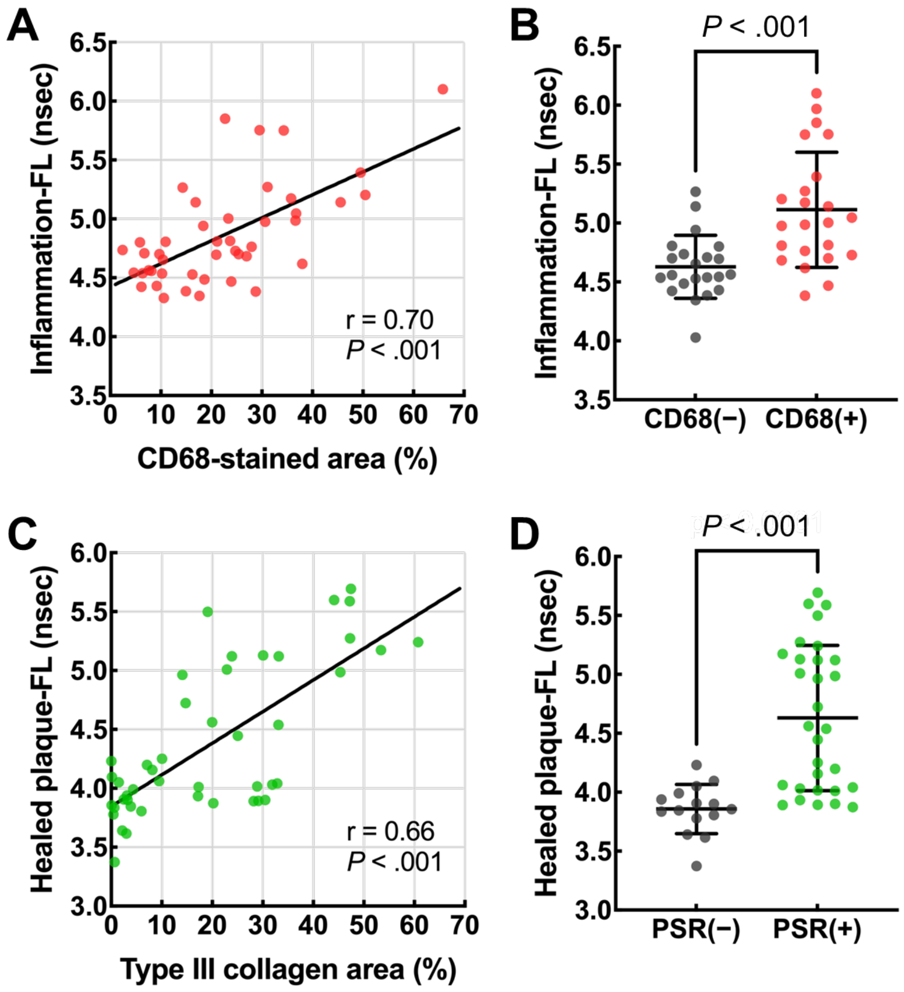

eFigure 8. Quantitative analysis of FLIm signals and histopathological stained area

(A, C) Correlative analysis of multispectral FLIm signals and histopathological staining. FLIm signals for inflammatory activity (inflammation-FL) and healed plaque burden (healed plaque-FL) correlated well with CD68 positive areas (A) and type III collagen fibers (C), respectively. **(B, D)** Quantitative comparative analysis of multispectral FLIm signals and histopathological staining. FLIm signals for inflammatory activity and healed plaque burden (inflammation- and healed plaque-FLs) showed significant differences with positive staining of CD68 macrophage accumulation (B) and type III collagen fibers (D), respectively. FL, fluorescence lifetime; PSR, picrosirius red.

© 2025 American Medical Association. All rights reserved, including those for text and data mining, AI training, and similar technologies.

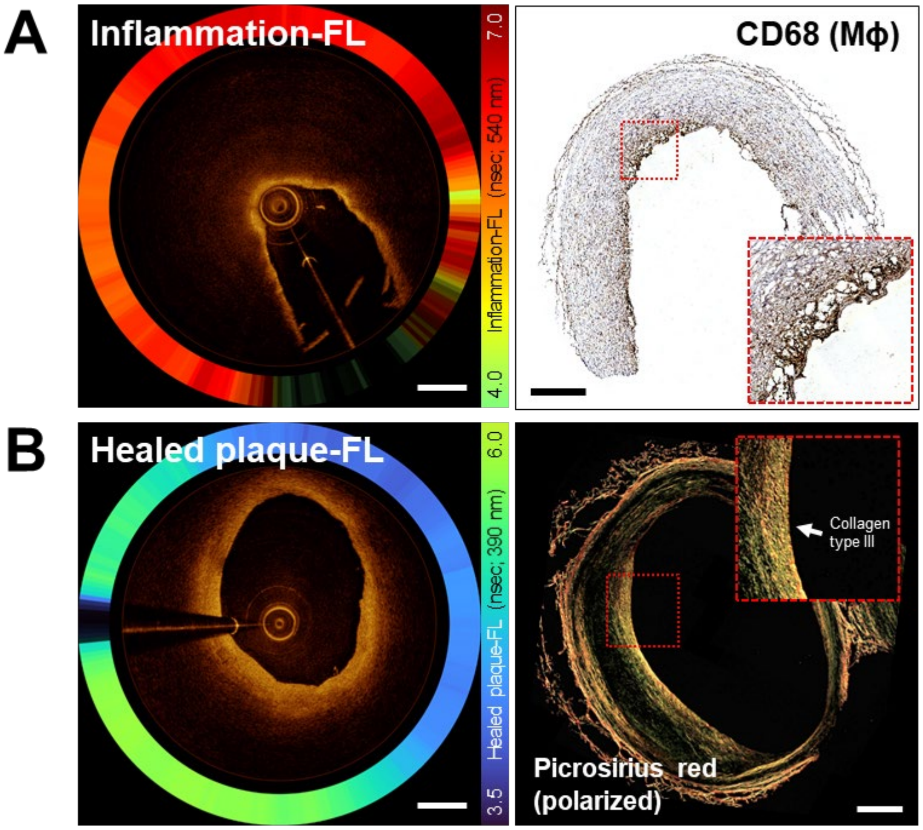

eFigure 9. Histopathological validation of OCT-FLIm images in varying human arterial specimens

(A) *Ex vivo* OCT-FLIm (left) and corresponding histopathologic image of macrophage immunostaining (CD68; right) obtained from a freshly resected human aorta. **(B)** *Ex vivo* OCT-FLIm (left) and corresponding histopathologic image of type III collagen fibers stained with picrosirius red (right) obtained from a freshly resected human renal artery. Scale bars indicate 1 mm for cross-sectional images. FL, fluorescence lifetime; MΦ, macrophages.

© 2025 American Medical Association. All rights reserved, including those for text and data mining, AI training, and similar technologies.

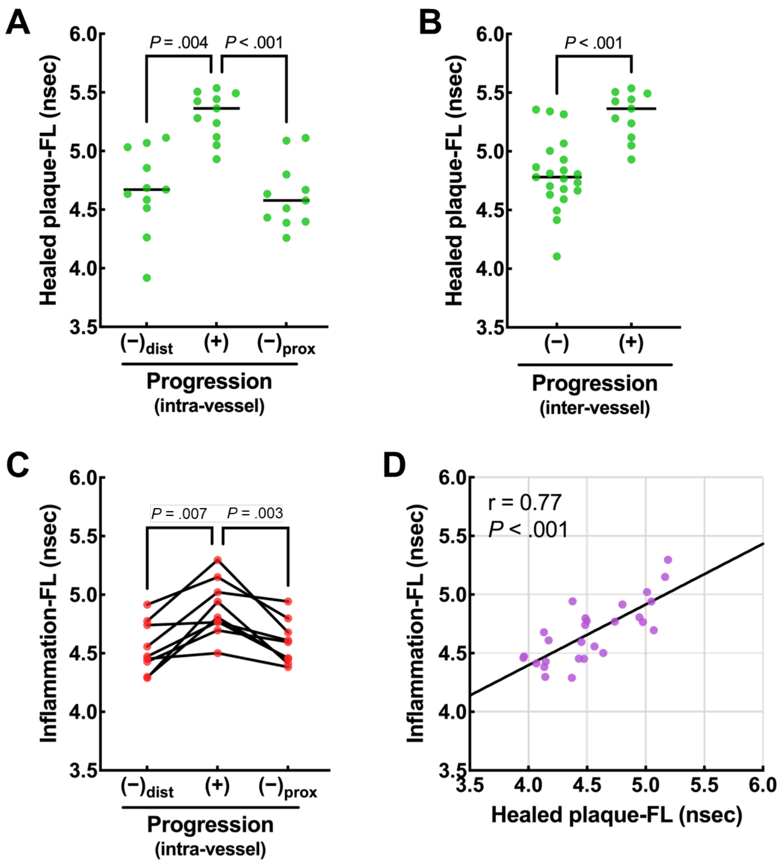

eFigure 10. Quantitative analysis of FLIm signals in target/culprit lesions of rapidly disease progression

(A, B) Intra- (A) and inter-vessel (B) analysis for quantitatively comparing healed plaque-FL measurements between rapid disease progression and non-progressive controls. Intra-vessel analysis shows significantly higher healed plaque-FL values in the target/culprit segments compared to proximal (5.31 ± 0.20 vs. 4.65 ± 0.28 nsec, *P* < .001) and distal segments (5.31 ± 0.20 vs. 4.67 ± 0.36 nsec, P = .004) within the same vessel. Inter-vessel analysis reveals elevated healed plaque-FL values in target/culprit plaques from patients with rapidly progressive coronary artery disease compared to non-target/non-culprit plaques in non-progressive control participants (5.31 ± 0.20 vs. 4.81 ± 0.30 nsec, *P* < .001). **(C)** Intra-vessel analysis for the quantitative comparison of inflammation-FL measurements between rapid disease progression and non-progressive controls. Rapidly progressive lesions showed concurrently elevated inflammatory FLIm signatures with the healed plaque burden in the target/culprit segments compared to proximal (target/culprit vs. proximal, inflammation-FL: 4.88 ± 0.24 vs. 4.59 ± 0.18 nsec, *P* = .007) and distal (target/culprit vs. distal, inflammation-FL: 4.88 ± 0.24 vs. 4.55 ± 0.22 nsec, *P* = .003) non-progressive segments. **(D)** Correlative analysis of multispectral FLIm signals within the segment of angiographic rapid disease progression. FLIm signals for healed plaque burden (healed plaque-FL) and inflammation (inflammation-FL) closely correlated along the target/culprit segment. The correlation between the multispectral FLIm signals were assessed using Spearman's rank correlation test. FL, fluorescence lifetime.

© 2025 American Medical Association. All rights reserved, including those for text and data mining, AI training, and similar technologies.

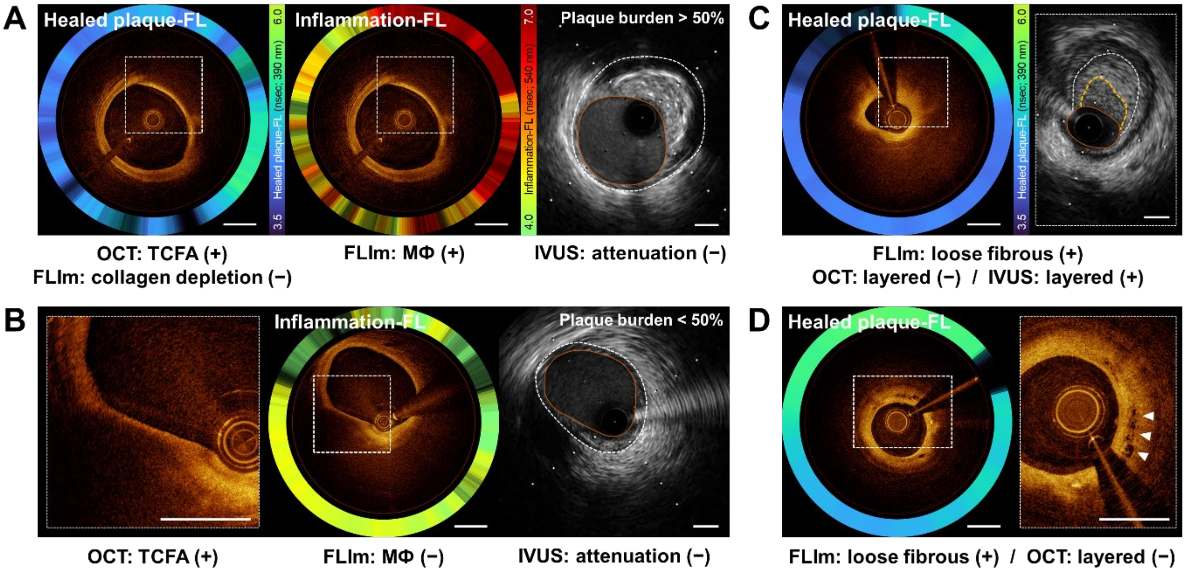

eFigure 11. OCT-FLIm images of thin-cap fibroatheroma-like lesions and atypical healed plaque morphologies

(A, B) Representative OCT images resembling thin-cap fibroatheromas (TCFAs) that did not meet the intravascular ultrasound (IVUS) criteria for TCFA, with differentiation enabled by complementary FLIm signals. (A) This lesion appears as TCFA in OCT but lacks attenuation in IVUS, indicating it is not a true TCFA. FLIm identified molecular signatures of heightened inflammation-FL but did not detect the shortened healed plaque-FL characteristics indicative of surface collagen depletion, typically observed in TCFA.[13] These findings suggest that the lesion is a fibrous plaque with superficial macrophage infiltration. (B) FLIm differentiated imaging artifacts, such as tangential signal dropout, from OCT morphologies resembling TCFA. Corresponding IVUS imaging confirmed the absence of a hypoechoic core at the identified location. **(C, D)** Representative images of atypical healed plaque layers in OCT. (C) FLIm identified healed plaque composition in a large plaque with a layered border located deep beyond OCT penetration, as confirmed by IVUS (yellow dashed contour). (D) FLIm visualized healed plaque composition in the presence of intraplaque micro-vessels (white arrowheads in the magnified view), which often obscure the layered structure. FL, fluorescence lifetime.

© 2025 American Medical Association. All rights reserved, including those for text and data mining, AI training, and similar technologies.

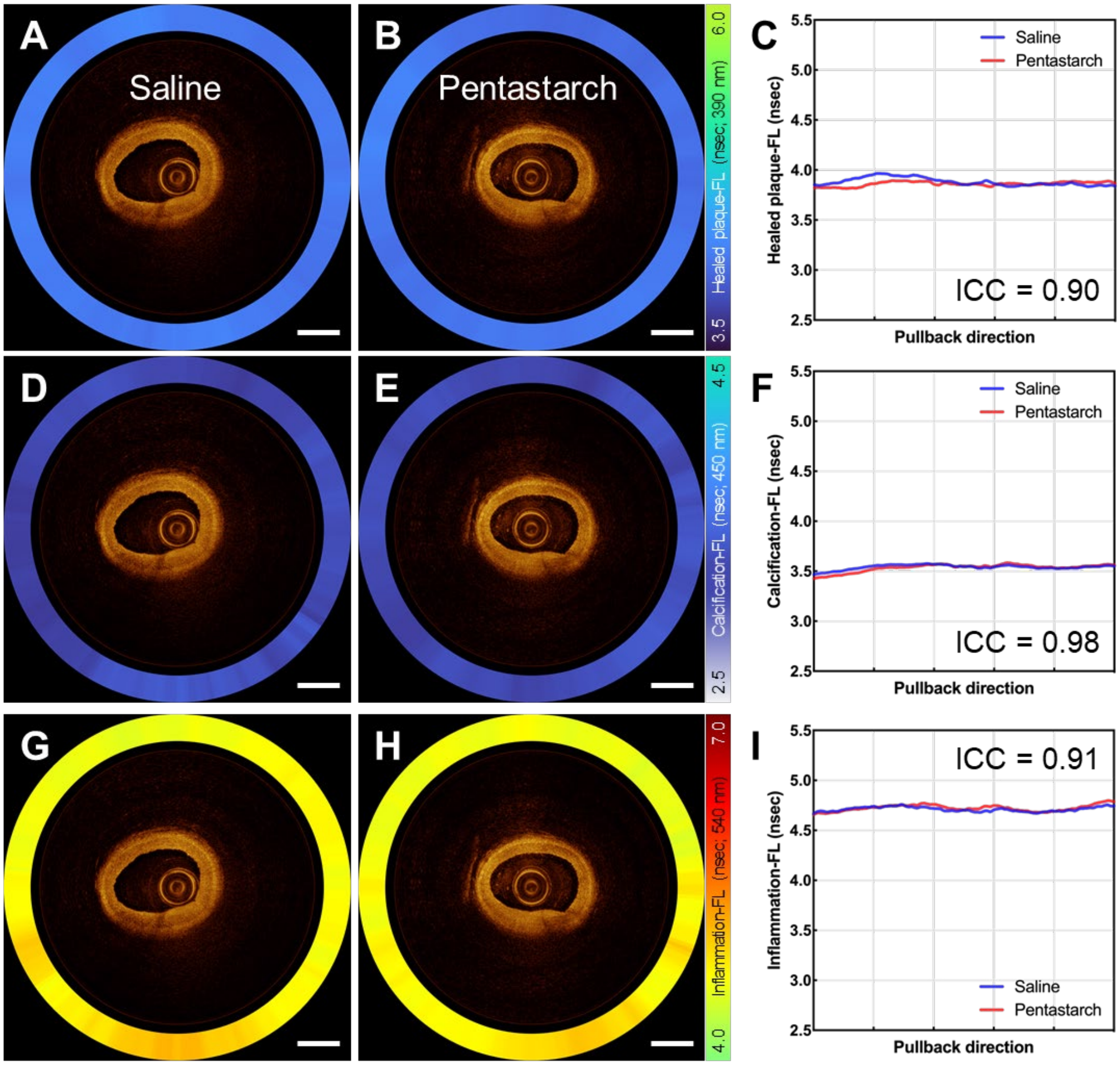

eFigure 12. Comparison of OCT-FLIm images obtained using the different solutions

(A-H) Multispectral OCT-FLIm images obtained using normal saline (A, D, G) vs. pentastarch (B, E, H). **(C, F, I)** Per-frame averaged fluorescence lifetime measurements from each spectral band along the pullback direction. Scale bars indicate 1 mm. FL, fluorescence lifetime; ICC, intraclass correlation coefficient.

© 2025 American Medical Association. All rights reserved, including those for text and data mining, AI training, and similar technologies.

eReferences

1. Kim S, Nam HS, Lee MW, et al. Comprehensive Assessment of High-Risk Plaques by Dual-Modal Imaging Catheter in Coronary Artery. *JACC Basic Transl Sci.* 2021;6(12):948-960. doi:10.1016/j.jacbts.2021.10.005

2. Institute ANS. *American National Standard for Safe Use of Lasers in Health Care.* Laser Institute of America; 2018.

3. Nam HS, Kang WJ, Lee MW, et al. Multispectral analog-mean-delay fluorescence lifetime imaging combined with optical coherence tomography. *Biomed Opt Express.* 2018;9(4):1930-1947. doi:10.1364/BOE.9.001930

4. Tearney GJ, Regar E, Akasaka T, et al. Consensus standards for acquisition, measurement, and reporting of intravascular optical coherence tomography studies: a report from the International Working Group for Intravascular Optical Coherence Tomography Standardization and Validation. *J Am Coll Cardiol.* 2012;59(12):1058-1072. doi:10.1016/j.jacc.2011.09.079

5. Mintz GS, Nissen SE, Anderson WD, et al. American College of Cardiology Clinical Expert Consensus Document on Standards for Acquisition, Measurement and Reporting of Intravascular Ultrasound Studies (IVUS). A report of the American College of Cardiology Task Force on Clinical Expert Consensus Documents. *J Am Coll Cardiol.* 2001;37(5):1478-1492. doi:10.1016/s0735-1097(01)01175-5

6. Shimokado A, Matsuo Y, Kubo T, et al. In vivo optical coherence tomography imaging and histopathology of healed coronary plaques. *Atherosclerosis.* 2018;275:35-42. doi:10.1016/j.atherosclerosis.2018.05.025

7. Otsuka F, Joner M, Prati F, Virmani R, Narula J. Clinical classification of plaque morphology in coronary disease. *Nat Rev Cardiol.* 2014;11(7):379-389. doi:10.1038/nrcardio.2014.62

8. Vergallo R, Crea F. Atherosclerotic Plaque Healing. *N Engl J Med.* 2020;383(9):846-857. doi:10.1056/NEJMra2000317

9. Fujii K, Hao H, Shibuya M, et al. Accuracy of OCT, grayscale IVUS, and their combination for the diagnosis of coronary TCFA: an ex vivo validation study. *JACC Cardiovasc Imaging.* 2015;8(4):451-460. doi:10.1016/j.jcmg.2014.10.015

© 2025 American Medical Association. All rights reserved, including those for text and data mining, AI training, and similar technologies.

10. Varghese F, Bukhari AB, Malhotra R, De A. IHC Profiler: an open source plugin for the quantitative evaluation and automated scoring of immunohistochemistry images of human tissue samples. *PloS one.* 2014;9(5):e96801
11. Kim JH, Song JW, Kim YH, et al. Multimodal Imaging-Assisted Intravascular Theranostic Photoactivation on Atherosclerotic Plaque. *Circ Res.* 2024;135(5):e114-e132
12. Zouridakis EG, Schwartzman R, Garcia-Moll X, et al. Increased plasma endothelin levels in angina patients with rapid coronary artery disease progression. *Eur Heart J.* 2001;22(17):1578-1584. doi:10.1053/euhj.2000.2588
13. Fatakdawala H, Gorpas D, Bishop JW, et al. Fluorescence Lifetime Imaging Combined with Conventional Intravascular Ultrasound for Enhanced Assessment of Atherosclerotic Plaques: an Ex Vivo Study in Human Coronary Arteries. *J Cardiovasc Transl Res.* 2015;8(4):253-263. doi:10.1007/s12265-015-9627-3
14. Barlis P, Gonzalo N, Di Mario C, et al. A multicentre evaluation of the safety of intracoronary optical coherence tomography. *EuroIntervention.* 2009;5(1):90-95. doi:10.4244/eijv5i1a14
15. Nam HS, Kang WJ, Lee MW, et al. Multispectral analog-mean-delay fluorescence lifetime imaging combined with optical coherence tomography. 2018;9(4):1930-1947
16. Kang DO, Nam HS, Kim S, Yoo H, Kim JW. Feasibility and safety of non-contrast optical coherence tomography imaging using hydroxyethyl starch in coronary arteries. *Sci Rep.* 2023;13(1):13818. doi:10.1038/s41598-023-40363-7

© 2025 American Medical Association. All rights reserved, including those for text and data mining, AI training, and similar technologies.
