# Supplementary material for: Intracoronary Structural-Molecular Imaging for Multitargeted Characterization of High-Risk Plaque: First-in-Human OCT-FLIm
Source: JAMA Cardiol. 2025 May 7;10(7):708–17. doi: 10.1001/jamacardio.2025.0928 (PMC12242695; doi:10.1001/jamacardio.2025.0928)
Supplement: Supplement 2. — Data Sharing Statement [file jamacardiol-e250928-s002.pdf]

Data Sharing Statement

Kim. Intracoronary Structural-Molecular Imaging for Multitargeted Characterization of High-Risk Plaque. *JAMA Cardiol*. Published May 07, 2025. doi:10.1001/jamacardio.2025.0928

Data
Data available: Yes
Data types: Other (please specify)
Additional Information: Raw data can be made accessible for research purposes upon reasonable request to the corresponding authors, contingent upon approval from the IRB at KUGH (Korea University Guro Hospital)
How to access data: Raw data can be made accessible for research purposes upon reasonable request to the corresponding authors, contingent upon approval from the IRB at KUGH (Korea University Guro Hospital)
When available: With publication

Supporting Documents
Document types: None

Additional Information
Who can access the data: Raw data can be made accessible for research purposes upon reasonable request to the corresponding authors, contingent upon approval from the IRB at KUGH (Korea University Guro Hospital)
Types of analyses: Deidentified raw data cand be made accessible for research purposes upon reasonable request to the corresponding authors, contingent upon approval from the IRB at KUGH (Korea University Guro Hospital)
Mechanisms of data availability: After approval of a proposal to the corresponding authors
Any additional restrictions: Additional approval from the IRB at KUGH (Korea University Guro Hospital) is needed.
